# Global burden, temporal trends and geographic disparities of younger-onset atrial fibrillation in adults aged 30–45 years, 1990–2021: a population-based study

**DOI:** 10.3389/fcvm.2025.1677005

**Published:** 2025-10-20

**Authors:** Jiayi Sun, Jiayang Dong, Lifeng Liang, Zuochen Xue, Jun Ding

**Affiliations:** ^1^ Department of Cardiology, Tianjin Chest Hospital, Tianjin, China; ^2^ Department of Cardiology, Tianjin Medical University General Hospital, Tianjin, China; ^3^ Department of Cardiology, Cardiovascular Center, Beijing Friendship Hospital, Capital Medical University, Beijing, China

**Keywords:** younger-onset atrial fibrillation, global burden of disease, temporal trends, geographic disparities, disability-adjusted life year

## Abstract

**Background:**

This study aimed to estimate the global burden, temporal trends, and geographic disparities of younger-onset atrial fibrillation (AF) in adults aged 30–45 years across 204 countries from 1990 to 2021, using data from the Global Burden of Disease Study. Focusing on this under-researched age group, we aimed to characterize the epidemiology of premature AF and its implications for prevention strategies.

**Methods:**

We analyzed the global burden of younger-onset AF (30–45 years) from 204 countries using Global Burden of Disease Study 2021 data. The primary outcomes included age-standardized prevalence, mortality, disability-adjusted life years (DALYs), and average annual percentage change (AAPC).

**Results:**

From 1990 to 2021, the age-standardized prevalence of AF among young and middle-aged adults decreased slightly from 57.81 to 57.48 cases per 100,000 population, with an AAPC of 0 (95% CI −0.05 to 0.05). Age-standardized mortality rates (ASMR) increased from 0.04 to 0.05 per 100,000 population, with an AAPC of 0.17% (95% CI 0.08 to 0.26). The age-standardized DALYs rate (ASDR) rate rose from 6.98 to 7.08 per 100,000 population (AAPC 0.05%, 95% CI −0.02 to 0.11). Men exhibited higher AF-related mortality, with an AAPC of 0.3% (95% CI 0.14 to 0.47). Higher Socio-demographic Index (SDI) countries showed higher AF prevalence (e.g., 111.54 per 100,000 in Australasia) but lower mortality rates, whereas low-SDI countries had higher mortality rates (AAPC 0.49% for low-middle SDI). Regional analysis revealed the highest prevalence increase in East Asia (AAPC 0.72%).

**Conclusions:**

From 1990 to 2021, the global age-standardized prevalence and ASDR for AF in adults aged 30–45 remained stable, though ASMR slightly increased, particularly among men and in regions with lower SDI. These findings emphasize the need for targeted prevention strategies to reduce AF-related mortality in this population.

## Introduction

Atrial Fibrillation (AF), a prevalent type of arrhythmia globally, significantly elevates the risk of major cardiovascular and cerebrovascular events. According to data from the Global Burden of Disease (GBD) study, the total number of AF patients has exceeded 59.7 million, accounting for 33% of all arrhythmia-related hospitalizations (1). Studies have projected that by 2060, the prevalence of AF among those aged 55 and above in Europe will increase from 8.8 million in 2010 to 17.9 million (2). In the United States, it is projected that the prevalence of AF will increase to 12.1 million by 2030 (3). This upward trend not only poses health challenges but is also accompanied by a significant economic burden. In the United States, direct-cost estimates per-patient-year ranged from $2,000 to $14,200, while in Europe, they ranged from €450 to €3,000 (4).

The pathological mechanism of atrial fibrillation (AF) is complex, and its core harm is mainly manifested as systemic embolism. Notably, AF in younger individuals exhibits distinct pathogenic mechanisms, with vagus-mediated pathways, high-intensity exercise, alcohol consumption, obesity, congenital heart disease, and genetic mutations serving as significant predisposing factors (5–8). Studies have shown that approximately 20%–30% of ischemic stroke events are caused by AF (9, 10). On the other hand, an increase in AF burden is associated with an increased risk of heart failure hospitalization and death (11). However, most countries and regions around the world lack comprehensive data on the burden of AF in young and middle-aged people. The disease burden of younger-onset AF may be severely underestimated. More seriously, due to a longer expected survival time, the lifetime cumulative risk of complications (such as dementia and kidney injury) in younger-onset AF patients may be higher than that in the elderly AF population (12, 13). From a socioeconomic perspective, AF patients may experience a decline in quality of life and occupational ability due to problems such as symptomatic palpitations and fatigue (14, 15). However, currently published clinical studies and guidelines focus more on the elderly population, resulting in a lack of evidence-based data and standardized intervention pathways for disease management in young and middle-aged people. Therefore, regular assessment of the epidemiological characteristics of AF in young and middle-aged people is crucial for formulating effective prevention and control strategies.

Therefore, our study analyzed the burden of AF among young and middle-aged adults from an epidemiological perspective. We investigated and analyzed the prevalence, mortality, and disability-adjusted life years (DALYs) of AF among adults aged 30–45 years at the global, regional, and national levels from 1990 to 2021, stratified by the level of social development, age, and gender of the young and middle-aged population.

## Methods

### Study population and data collection

This study utilized the 2021 GBD database—which employs three standardized tools: Cause of Death Ensemble Model (CODEm), Spatiotemporal Gaussian Process Regression (ST-GPR), and Disease Model-Bayesian Meta-regression (DisMod-MR)—to analyze burden trends of younger-onset AF from 1990 to 2021 (Supplementary Methods). The database is mainly used for disease burden analysis, risk factor research, comparison of regional health inequalities, time trend analysis, health policy evaluation, and assessment of the health status of specific populations. It covers 306 diseases and injuries as well as 87 risk factors. The study population consisted of AF patients aged 30–45 years. We extracted data on AF among individuals aged 30–45 years from the 2021 GBD study, including prevalence specific to location, age, and sex; mortality; numbers and rates of DALYs; and risk factor-attributable DALYs [with corresponding 95% uncertainty interval (UI)]. DALYs measure the health loss in a population over a specific time period due to disease or injury, mainly composed of years of life lost (YLL) and years lived with disability (YLD).

We evaluated the burden of AF at the global, regional (21 regions), and national (204 countries) levels, with a particular focus on the young and middle-aged population aged 30–45 years. The analysis divided this population into three age groups: 30–34 years, 35–40 years, and 41–45 years. In addition, this study conducted a stratified analysis based on the Socio-demographic Index (SDI). The evaluation criteria for the SDI include the per capita income level (reflecting material resources and medical accessibility), the average years of education of the population aged 15 and above (reflecting health literacy and disease prevention ability), and the total fertility rate of women under 25 years of age (representing population growth pressure and the burden of resource allocation). The SDI values range from 0 to 1, representing the economic development level from low to high. Countries and regions globally are divided into five levels according to the SDI: low SDI, low-middle SDI, middle SDI, high-middle SDI, and high SDI. This stratified design can help us reveal the association between the disease burden and the regional development level.

### Statistical analysis

This study analyzed the burden of AF among young and middle-aged adults aged 30–45 years globally. Age-standardized prevalence, mortality, and DALY rates (per 100,000 population) were compared across age groups, genders, regions, and countries. To address age-structure heterogeneity, age-standardized rates with corresponding 95% confidence interval (CI) were calculated using weighted averages, enabling valid inter-regional comparisons. Prevalence represents the proportion of existing cases within a specified timeframe. Mortality directly quantifies AF-attributable deaths, serving as a critical severity indicator. Where AF does not cause direct fatality, it may substantially reduce quality of life and increase disability burden. Thus, DALYs comprehensively capture disease impact on health status. The average annual percentage change (AAPC) was estimated using joinpoint regression to assess temporal trends. AAPC represents the mean annualized change rate derived from weighted slope coefficients of the joinpoint model (1990–2021). An upward trend is established when both AAPC and the lower 95% CI limit exceed zero; conversely, a downward trend is confirmed when both AAPC and the upper 95% CI limit fall below zero. Calculate the cumulative absolute change (per 100,000) (*Δ*R) of age—standardized rates from 1990 to 2021 and its 95% confidence interval to evaluate the changes in age—standardized rates.

All analyses were performed using R v4.4.1, with trend analysis conducted in Joinpoint v5.1.0.0. Statistical significance was defined as *P* < 0.05.

## Results

### Global trends

Globally during 1990–2021, the number of young and middle-aged patients (aged 30–45 years) with AF increased from 565,407 to 952,298, representing a 68% increase. However, the age-standardized prevalence did not change significantly. The age-standardized prevalence decreased from 57.81 cases per 100,000 in 1990 to 57.48 cases per 100,000 in 2021. During these 31 years, the AAPC in prevalence was 0 (Table 1). In addition, compared with the total number of AF patients, the proportion of young and middle-aged AF patients showed a gradually increasing and then stabilizing trend during 1990–2000, and a gradually decreasing trend during 2001–2021 (Supplementary Figure S1A). The age-standardized mortality rate (ASMR) for AF in young and middle-aged populations increased from 0.04 cases per 100,000 in 1990 to 0.05 cases per 100,000 in 2021, with an AAPC of 0.17% (Supplementary Table S1). The age-standardized DALY rate (ASDR) in this population increased slightly from 6.98 cases per 100,000 in 1990 to 7.08 cases per 100,000 in 2021, and the AAPC change trend was not obvious (AAPC 0.05%, 95%CI −0.02 to 0.11) (*Δ*R 0.01, 95%CI −0.20 to 0.40) (Supplementary Table S2). However, compared with total AF cases, the proportions of deaths and DALYs related to AF among individuals aged 30–45 years both showed gradually decreasing trends (Supplementary Figures S1B, S1C).

**Table 1:** Age-standardized prevalence and AAPC of AF among people aged 30-45 years at the global and regional levels from 1990 to 2021.

Location	Prevalence (95% UI)					
	Cases in 1990	Age standardised rate in 1990 (per 100 000)	Cases in 2021	Age standardised rate in 2021 (per 100 000)	AAPC (95% CI)	*Δ*R (95% CI)
Global	565,407.36 (305,244.27–943,342.76)	57.81 (31.34–96.3)	952,298.03 (552,454.39–537,465.3)	57.48 (33.36–92.78)	0 (−0.05–0.05)	−0.33 (−62.94–1.44)
Sex:						
Female	201,741 (101,986.97–348,540.83)	42.04 (21.36–72.48)	360,285.82 (195,004.04–03,399.68)	43.87 (23.75–73.45)	0.16 (0.12–0.21)	1.83 (−48.72–52.09)
Male	363,666.36 (199,658.22–596,267.39)	72.98 (40.21–119.47)	592,012.21 (352,258.29–33,821.68)	70.85 (42.18–111.74)	−0.08 (−0.14–−0.02)	−2.73 (−77.29–39.53)
Age group (years):						
30–34	25,623.11 (12,183.14–44,677.75)	6.65 (3.16–11.59)	39,718.53 (20,426.7–67,236.8)	6.57 (3.38–11.12)	−0.01 (−0.06–0.03)	−0.08 (−8.21–7.96)
35–39	162,148.72 (77,063.99–282,497.22)	46.03 (21.88–80.2)	256,344.4 (132,417.62–32,320.28)	45.71 (23.61–77.08)	−0.01 (−0.05–0.04)	−0.32 (−56.59–55.20)
40–45	377,635.52 (215,997.14–616,167.79)	131.82 (75.4–215.08)	656,235.11 (399,610.06–1,037,908.22)	131.18 (79.88–207.48)	0 (−0.05–0.05)	−0.64 (−135.20–132.08)
SDI level:						
High	181,425.04 (104,764.03–287,592.22)	88.04 (50.8–139.63)	225,285.47 (156,156.45–13,660.76)	92.42 (63.96–128.77)	0.18 (0.12–0.24)	4.38 (−75.67–88.97)
High-middle	126,794.14 (67,248.79–212,866.11)	58.65 (31.29–98.26)	193,525.34 (108,265.29–13,253.14)	63.72 (35.63–103.16)	0.28 (0.23–0.34)	5.07 (−62.63–71.87)
Middle	161,755.71 (84,844.4–275,147.81)	53.17 (28.05–90.18)	309,043.58 (165,255.68–21,947.26)	56.6 (30.28–95.58)	0.21 (0.1–0.32)	3.43 (−59.90–67.53)
Low-middle	70,698.81 (35,456.34–123,089.02)	38.96 (19.63–67.67)	158,278.48 (80,089.96–277,697.88)	41 (20.79–71.87)	0.18 (0.12–0.24)	2.04 (−46.88–52.24)
Low	24,171.43 (11,915.36–42,506.43)	34.96 (17.35–61.26)	65,340.77 (32,748.81–114,735.88)	37.21 (18.72–65.2)	0.19 (0.18–0.21)	2.25 (−42.54–47.85)

AAPC, average annual percentage change; CI, confidence interval; SDI, socio-demographic index; UI, uncertainty interval; *Δ*R, Absolute change in age-standardized rate (per 100,000 population).

### Global trends by sex

Globally during 1990–2021, among the young and middle-aged population aged 30–45 years, the age-standardized prevalence of AF increased in women (from 42.04 cases per 100,000 in 1990 to 43.87 cases per 100,000 in 2021, with an AAPC of 0.16%, 95%CI 0.12 to 0.21) (*Δ*R 1.83, 95%CI −48.72 to 52.09) (Table 1). In men, the age-standardized prevalence of AF decreased (from 72.98 cases per 100,000 in 1990 to 70.85 cases per 100,000 in 2021, with an AAPC of −0.08%, 95%CI −0.14 to −0.02) (*Δ*R −2.73, 95%CI−77.29 to 39.53) (Table 1). During the same period, the ASMR of AF in young and middle-aged men increased (AAPC 0.3%, 95%CI 0.14 to 0.47) (ΔR 0, 95%CI −0.03 to 0.03) (Supplementary Table S1), while the ASMR trend for women in this population was not significant (AAPC 0.03%, 95%CI −0.06 to 0.12) (*Δ*R 0, 95%CI −0.02 to 0.02) (Supplementary Table S1).

For DALYs, the increase was more pronounced in men (from 5.31 cases per 100,000 in 1990 to 5.48 cases per 100,000 in 2021, AAPC 0.11%, 95%CI 0.05 to 0.17) (*Δ*R 0.17, 95%CI −0.10 to 0.44) than in women (from 8.58 cases per 100,000 in 1990 to 8.65 cases per 100,000 in 2021, AAPC 0.02%, 95%CI −0.05 to 0.09) (*Δ*R 0.07, 95%CI −0.17 to 0.31) (Supplementary Table S2). This gender disparity persisted irrespective of SDI variations. Globally, the disease burden remained higher in men than in women, particularly in regions with middle or lower SDI, where the difference was most evident (Supplementary Figures S2, S3).

### Global trends by age subgroup

Globally during 1990–2021, AF prevalence in all age subgroups (30–34 years, 35–39 years, 40–45 years) of young and middle-aged individuals showed an upward trend (30–34 years: 25,623 to 39,718; 35–39 years: 162,148 to 256,344; 40–45 years: 377,635 to 656,235). Notably, crude prevalence growth was higher in women than men across subgroups (Supplementary Figure S4). However, age-standardized AF prevalence trends remained non-significant (Table 1). During this period, ASMR increased in the 35–39 years and 40–45 years subgroups, most notably in 35–39 years ((AAPC 0.32%, 95%CI 0.24 to 0.39) vs. (AAPC 0.14%, 95%CI 0.03 to 0.25) in 40–45 years). The 30–34y subgroup showed no significant ASMR trend (Supplementary Table S1). In 2021, ASMR increased with age (30–34 years: 0.02 cases per 100,000; 40–45 years: 0.09 cases per 100,000) (Supplementary Table S1). Consistently, men showed rising mortality trends vs. women in all age subgroups (Supplementary Figure S4).

From 1990 to 2021, ASDR for AF in the 35–39 years age subgroup showed the most pronounced upward trend (AAPC 0.08%, 95%CI 0.02 to 0.14) (*Δ*R 0.12, 95%CI −0.11 to 0.35) compared to the 30–34 years and 40–45 years subgroups (Supplementary Table S2).

### Global trends by sociodemographic index

Between 1990 and 2021, age-standardized AF prevalence among individuals aged 30–45 years increased across all SDI subgroups, particularly in high-middle SDI countries (AAPC 0.28%,95%CI 0.23 to 0.34) (*Δ*R 5.07, 95%CI −62.63 to 71.87). In 2021, high SDI countries had the highest AF prevalence (92.4 cases per 100 000) in this population (Table 1). During this period, AF-related mortality increased in low, low-middle, and middle SDI subgroups, with the largest increase in low-middle SDI (AAPC 0.49%, 95%CI 0.37 to 0.61) (*Δ*R 0.01 95%CI −0.02 to 0.04). Conversely, high-middle SDI subgroups showed declining mortality (AAPC −0.47%, 95%CI −0.74 to −0.19) (*Δ*R 0 95%CI −0.02 to 0.02) (Supplementary Table S1). When SDI exceeded 0.625, this declining trend became more pronounced (Supplementary Figure S5). In 2021, high-middle SDI countries had the lowest ASMR (0.03 cases per 100,000) (Figures 2, 3, Supplementary Table S1).

During the same period, ASDR showed significant increases across all SDI subgroups, most notably in low-middle SDI countries (AAPC 0.3%, 95%CI 0.18 to 0.42) (ΔR 0.52 95%CI −0.10 to 1.14). However, in 2021, high SDI countries had the highest ASDR (9.95 cases per 100,000) (Supplementary Table S2).

### Regional trends

During 1990–2021, Australasia was the only region among 21 studied demonstrating a declining trend in AF prevalence among adults aged 30–45 years (AAPC −0.95%, 95%CI −1.09 to −0.81) (*Δ*R −33.27 95%CI −66.06 to −0.48) (Supplementary Table S3). The most rapid increases in age-standardized AF prevalence occurred in East Asia (AAPC 0.72%,95%CI 0.66 to 0.78) (*Δ*R 13.47 95%CI −0.40 to 27.34), High-income Asia Pacific (AAPC 0.59%, 95%CI 0.2 to 0.99) (*Δ*R 16.47 95%CI 1.88 to 31.61), and Central Europe (AAPC 0.41%, 95%CI 0.33 to 0.49) (*Δ*R 7.22 95%CI −0.61 to 15.05) (Supplementary Figure S6). In 2021, Australasia exhibited the highest age-standardized AF prevalence (111.54 cases per 100,000 population) (Supplementary Table S3). Gender-stratified analysis identified High-income Asia Pacific had the highest female prevalence (83.05 cases per 100,000), while Western Europe had the highest male prevalence (174.36 cases per 100,000) (Supplementary Table S4).

During 1990–2021, High-income North America showed the largest increase in ASMR of AF among adults aged 30–45 years (AAPC 1.82%, 95%CI 1.55–2.08) (*Δ*R 0.03 95%CI 0.02–0.03), while Central Europe exhibited the most pronounced decline (AAPC −1.71%,95%CI −1.97 to −1.45) (*Δ*R −0.02 95%CI −0.02 to −0.01) (Supplementary Table S5). Concurrently, the steepest rises in ASDR occurred in East Asia (AAPC 0.45%, 95%CI 0.41–0.5) (*Δ*R 0.86 95%CI −6.01 to 7.99) and High-income North America (AAPC 0.45%, 95%CI 0.34–0.56) (*Δ*R 1.41 95%CI −7.26 to 8.59), contrasting with Australasia's significant decrease (AAPC −0.79%, 95%CI −0.86 to −0.72) (*Δ*R −2.59 95%CI −14.22 to 7.67) (Supplementary Table S6, Figure S6). Sex stratification did not alter these trends (Supplementary Figure S7). In 2021, Oceania had both the highest ASMR (0.35 cases per 100,000) and ASDR (22.88 cases per 100,000) for AF in this cohort (Supplementary Tables S5, S6). No significant sex-based differences were observed (Supplementary Table S4).

### National trends

**Figure 1:**
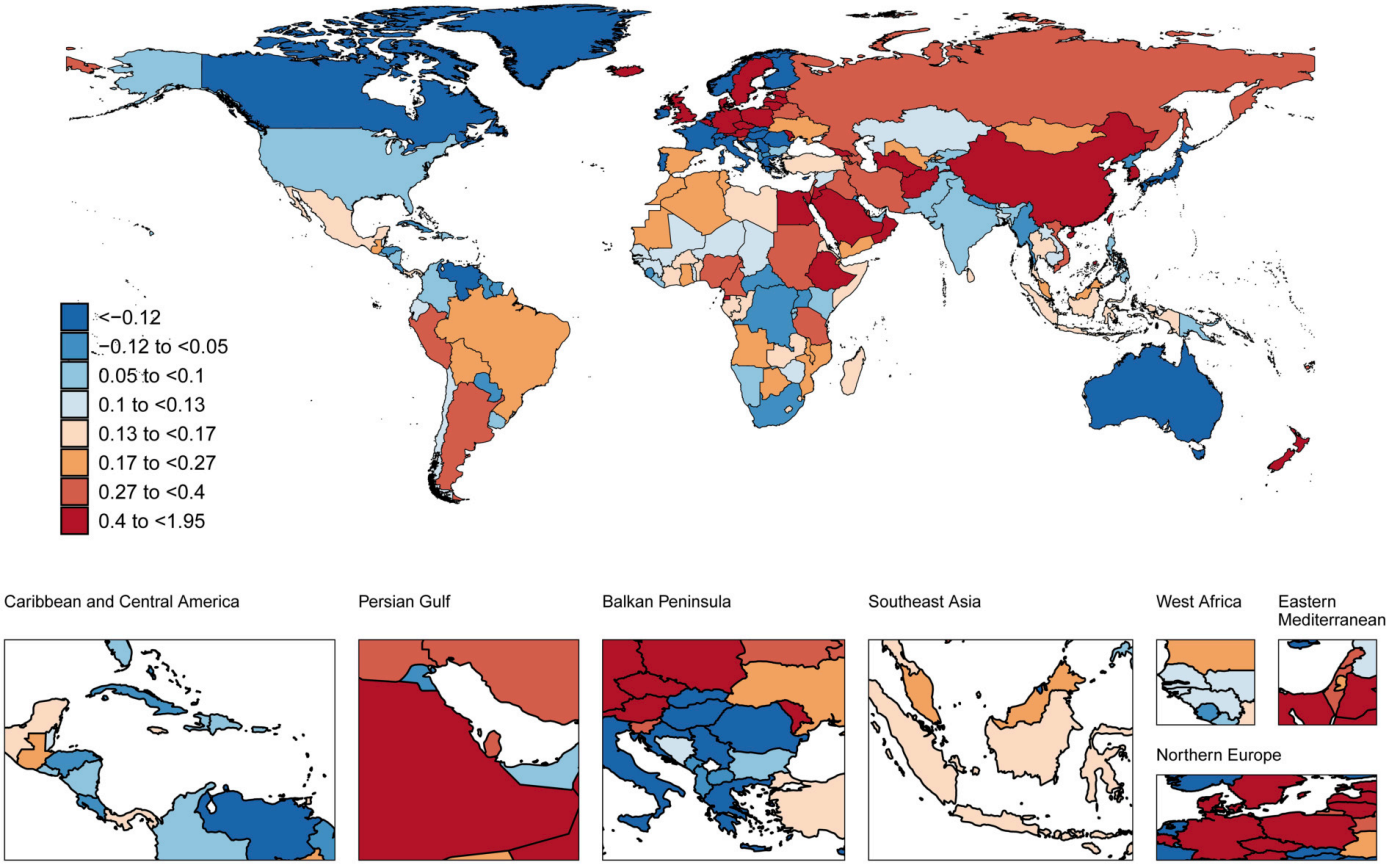
Global spatial distribution of atrial fibrillation prevalence trends: AAPC among adults aged 30-45 years, 1990-2021. World map adapted from: “Global country administrative boundary data”, Research and Environmental Science Data Platform (https://www.resdc.cn/).

**Figure 2:**
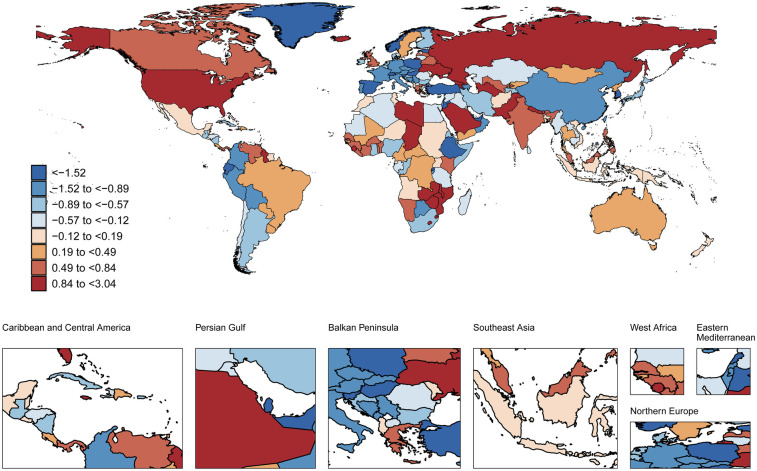
Global spatial distribution of atrial fibrillation ASMR trends: AAPC among adults aged 30-45 years, 1990-2021. World map adapted from: “Global country administrative boundary data”, Research and Environmental Science Data Platform (https://www.resdc.cn/).

**Figure 3:**
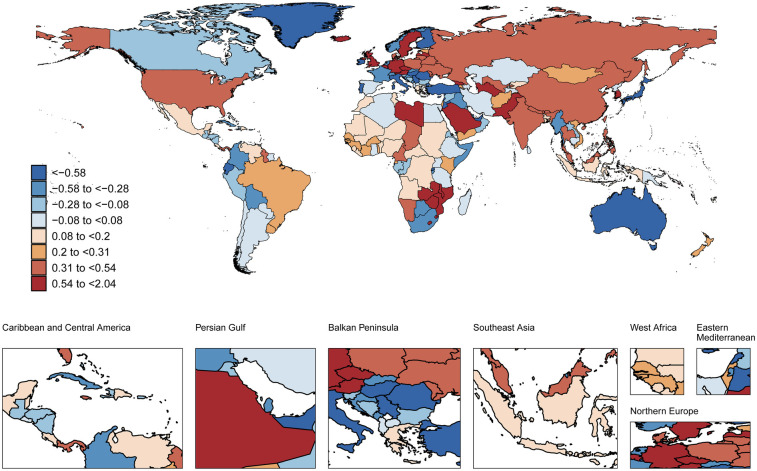
Global spatial distribution of atrial fibrillation ASDR trends: AAPC among adults aged 30-45 years, 1990-2021. World map adapted from: “Global country administrative boundary data”, Research and Environmental Science Data Platform (https://www.resdc.cn/).

At the national level (1990–2021), Belgium showed the steepest rise in age-standardized AF prevalence among adults aged 30–45 years (AAPC 1.95%, 95%CI 1.83–2.07), followed by the Republic of Korea (AAPC 1.67%, 95%CI 1.17–2.17) and Georgia (AAPC 1.64%, 95%CI 1.47–1.81). In contrast, Japan demonstrated the most significant decline in age-standardized AF prevalence (AAPC −1.19%, 95%CI −1.29 to −1.09). Concurrently, Georgia had the greatest increases in both ASMR (AAPC 3.04%, 95%CI 2.23–3.85) and ASDR (AAPC 2.04%, 95%CI 1.71–2.37), while Lebanon showed the sharpest ASMR reduction (AAPC −3.44%, 95%CI −3.79 to −3.08). In 2021, the Republic of Korea recorded the highest age-standardized AF prevalence (224.38 cases per 100,000). Nauru exhibited the highest ASMR (0.77 cases per 100,000) and ASDR (45.65 cases per 100,000) values (Figures 1–3 and Supplementary Table S7).

## Discussion

Globally during 1990–2021, AF cases among adults aged 30–45 years increased from 565,407 to 952,298 (68% rise). The age-standardized prevalence showed non-significant change, declining marginally from 57.81 to 57.48 per 100,000 population. Despite advances in cardiovascular risk management, the AF burden appeared to increase during the study period, as evidenced by positive annual percentage changes in both age-standardized mortality rate (AAPC 0.17%) and age-standardized disability-adjusted life years rate (AAPC 0.05%). Significant disparities in AF-related DALYs existed across SDI levels, with low-middle SDI countries exhibiting steeper ASDR increases (AAPC 0.30%). Effective rhythm control and complication prevention remain clinical challenges in this cohort. Our findings advance understanding of evolving global AF burdens by delineating incidence trends specifically in 30–45 years.

Current research reports show unequal prevalence rates of AF between men and women (16). Generally, the prevalence of AF is higher in men than in women (17). Our analysis reveals that although men with AF outnumbered women, age-standardized prevalence rose among women (AAPC 0.16%) but declined in men (AAPC −0.08%) during 1990–2021. Some research results suggest that reproductive factors have a causal effect on cardiovascular diseases in women. The higher the number of live births, the greater the risk of AF (18). Women are more affected by the impact of psychosocial stress on cardiovascular health than men, particularly during young and middle adulthood. Moreover, events such as menarche, pregnancy, and menopause may further amplify this stress (19, 20). However, ASMR and ASDR of AF in young and middle-aged men are significantly higher than in women, especially in low SDI regions. This sex-based difference may relate to physiological, lifestyle, and socioeconomic factors. Women may prioritize health management earlier in life and demonstrate stronger disease awareness and self-management capabilities, thereby reducing AF-related mortality. Conversely, men exhibit greater exposure to cardiovascular risk factors including smoking, alcohol consumption, and obesity, which may accelerate AF pathogenesis. Elevated body mass index (BMI) constitutes the strongest predictor of AF, with higher risk ratios in men vs. women. Moderate alcohol consumption elevates AF risk exclusively in men, whereas heavy intake increases risk in both sexes (21–23). Clinical studies have demonstrated that while estradiol reduces AF risk, sole administration of conjugated estrogens elevates this risk, suggesting complex interactions between hormone preparations and estrogen receptor specificity (24, 25). At the cellular level, cardiomyocytes express both estrogen and androgen receptors, indicating that sex hormones directly modulate ion channels and their expression profiles (26, 27). These electrophysiological alterations manifest as a net prolongation of action potential duration (APD) and QT interval by estrogen, whereas testosterone induces net APD/QT shortening—potentially explaining the divergent clinical outcomes (28). However, as current mechanistic evidence predominantly derives from ventricular cardiomyocyte studies, further confirmation is required regarding the translatability of these findings to atrial electrophysiological phenotypes. The precise mechanism underlying sex differences in AF patients remains incompletely characterized, though existing studies implicate cardiac structural, electrophysiological, and hormonal factors in AF pathogenesis (29).

Our analysis demonstrates rising AF prevalence across all age subgroups (30–45 years), with particularly pronounced increases in the 35–39 years and 40–45 years cohorts. Advancing age constitutes an independent risk factor for AF (30). Unlike elderly AF patients, younger individuals exhibit stronger risk factor associations with obesity and alcohol consumption (31). Notably, age-standardized prevalence changes remained non-significant across subgroups, potentially attributable to population aging and healthcare advancements. Mortality increased in both 35–39 years and 40–45 years subgroups, with the steepest rise observed in the 35–39 years cohort (AAPC 0.32%).

This indicates that the AF-related death risk among young and middle-aged people gradually increases with age, suggesting that we should strengthen the screening and intervention of AF in the early middle age.

The findings of this study reveal a substantial rise in the prevalence of AF in East Asia, with an AAPC of 0.72%. This increase may be linked to the accelerated aging process of the population and the growing prevalence of metabolic syndrome (32). Conversely, despite North America's leading position in global medical investment, the mortality rate of AF among individuals aged 30–45 shows an abnormal upward trend (AAPC 1.82%), further highlighting the “medical paradox” in regions with a high SDI. Specifically, the uneven distribution of social resources and over—intervention actually exacerbate the disease burden (33–35). In the future, targeted prevention and control strategies are required. East Asia should concentrate on metabolic intervention and utilize wearable devices for screening. Meanwhile, North America urgently needs to tackle medical inequality, establish a drug safety warning system, and standardize the comprehensive management process of AF.

Significant disparities exist in AF epidemiological characteristics across regions among adults aged 30–45 years. These variations may stem from genetic, environmental, lifestyle, and healthcare resource factors (36, 37). Our analysis demonstrates higher age-standardized AF prevalence but lower mortality in SDI regions, contrasting with lower prevalence yet higher mortality in low SDI areas. This pattern likely reflects superior healthcare resources and disease management capabilities in high SDI settings. In regions with a high SDI, the utilization rate of wearable intelligent detection devices among the population is high, such as smartwatches. Moreover, the population screening policies implemented by the universal healthcare systems in high-SDI regions (e.g., the UK's NHS) can detect more asymptomatic or occult subclinical AF patients. This detection gradient and systemic ascertainment bias may lead to an overestimation of the disease burden in high-SDI regions. These strategies—rigorous risk factor control, advanced diagnostics, and novel therapies—collectively improve quality of life and reduce AF mortality (20, 38, 39). Collectively, these observations highlight socioeconomic influences on early cardiovascular health, indicating that low SDI regions face challenges in healthcare access, health education, and cardiovascular risk prevention for younger populations (40).

Utilizing GBD study data, this analysis acknowledges inherent limitations despite the database's broad coverage. First, primary reliance on national reporting systems risks data omissions and ascertainment bias, with additional uncertainties arising from GBD model estimation methodologies. Second, substantial variability in primary data sources combined with geographic heterogeneity in AF diagnostic criteria and therapeutic approaches may compromise result accuracy. Third, temporal data latency reflecting time lags between clinical events and registry reporting potentially limits reflection of contemporary epidemiological profiles. Finally, this study considered the physiological changes and health risks of the population when stratifying age (such as, in the age group of 30–34 years, people in this stage begin to face work-life stress, and the proportion of anxiety increases, suggesting that both mental and physical health need to be concerned simultaneously. The risk of some chronic diseases (e.g., hypertension and hyperlipidemia) gradually emerges; in the age group of 40–45 years, women enter the perimenopausal period, with fluctuating estrogen levels and an increased risk of decreased bone density. The risk of cardiovascular and cerebrovascular diseases in men further rises, and the detection rates of chronic diseases such as arteriosclerosis and diabetes increase). However, there is currently a lack of specific literature or evidence to support this, and there may be grouping bias. There are limitations in calculating the ΔR. The measurement errors in the data of the first and last years may significantly widen the confidence interval of the absolute change. In addition, the study population consisted of young-onset AF patients aged 30–45 years, which is a small low-prevalence subgroup. It is possible that the AAPC is significant while the absolute change rate is not. Consequently, priority should be given to: (1) implementing real-time AF surveillance systems, (2) enhancing data collection from adults aged 30–45 years, and (3) refining statistical methodologies through approaches such as (a) sensitivity analysis, and (b) stratified exploration of sex-by-SDI and age-by-SDI differentials through decomposition analysis,. These measures will collectively reduce research uncertainty, improve result interpretability, and strengthen the validity of future studies.

This study constitutes critical evidence for developing targeted AF prevention strategies in adults aged 30–45 years. Implementation priorities include: (1) enhanced health education to improve AF disease literacy and self-management capacity; (2) early screening and intervention programs for high-risk subgroups (males and low sociodemographic index regions) to reduce AF incidence and mortality; (3) increased national investment in AF care infrastructure to enhance healthcare accessibility and quality; (4) strengthened international technical cooperation for resource-limited settings, and (5) For regions with different SDI levels, different public health strategies should be formulated. For example, in high-SDI areas, region-tailored strategies such as wearables screening can be implemented, while in low-SDI settings, the focus can be on hypertension detection. Our findings further necessitate personalized intervention protocols, optimized resource allocation, and age-specific clinical guidelines. These evidence-based recommendations hold significant implications for cardiovascular practice, epidemiological research, and management of younger-onset AF.

## Conclusion

This systematic evaluation delineates global epidemiological patterns of AF among adults aged 30–45 years, quantifying sex-specific, age-stratified, and SDI-driven disparities in disease burden across geographic regions. These findings establish critical evidence for investigating AF pathogenesis and optimizing targeted interventions, informing substantial public health initiatives worldwide.

## Data Availability

The datasets presented in this study can be found in online repositories. The names of the repository/repositories and accession number(s) can be found in the article/Supplementary Material.
