# Supplementary material for: Global burden, temporal trends and geographic disparities of younger-onset atrial fibrillation in adults aged 30–45 years, 1990–2021: a population-based study
Source: Front Cardiovasc Med. 2025 Oct 20;12:1677005. doi: 10.3389/fcvm.2025.1677005 (PMC12580189; doi:10.3389/fcvm.2025.1677005)
Supplement: Supplementary file 1 [file Supplementaryfile1.docx]

**Supplemental Material**

**Global Burden, Temporal Trends and Geographic Disparities of Younger-Onset Atrial Fibrillation in Adults Aged 30–45 Years, 1990–2021: A Population-Based Study**

**Content of Supplemental Material**

**Supplementary Methods**

1. **Data sources**
2. **Fatal disease modeling**
3. **Nonfatal burdens modeling**
4. **Processing of missing data**
5. **Data quantification**

**Supplementary Table**

**Supplementary Table 1.** Age-standardized mortality rates (ASMR) and average annual percentage change (AAPC) of atrial fibrillation among people aged 30 - 45 years at the global and regional levels from 1990 to 2021

**Supplementary Table 2.** Age-standardized disability-adjusted life years (ASDR) and AAPC of atrial fibrillation among people aged 30 - 45 years at the global and regional levels from 1990 to 2021

**Supplementary Table 3.** Age-standardized prevalence of atrial fibrillation and its AAPC among adults aged 30–45 years at regional levels, 1990–2021

**Supplementary Table 4.** Age-standardized prevalence, ASMR and ASDR of atrial fibrillation among adults aged 30–45 years, by sex and region, 2021

**Supplementary Table 5.** ASMR of atrial fibrillation and its AAPC among adults aged 30–45 years at regional levels, 1990–2021

**Supplementary Table 6.** ASDR of atrial fibrillation and its AAPC among adults aged 30–45 years at regional levels, 1990–2021

**Supplementary Table 7.** Age-standardized prevalence, ASMR and ASDR of atrial fibrillation in adults aged 30–45 years in 2021 and their AAPCs between 1990-2021 in 204 countries and territories

**Supplementary Figure**

**Figure S1.** (A) Change in the proportion of prevalence among atrial fibrillation patients aged 30-45 years relative to all atrial fibrillation patients, 1990 to 2021. (B) Change in the proportion of deaths among atrial fibrillation patients aged 30-45 years relative to all atrial fibrillation deaths, 1990 to 2021. (C) Change in the proportion of DALYs among atrial fibrillation patients aged 30 - 45 years relative to all atrial fibrillation DALYs, 1990 to 2021.

**Figure S2.** Temporal trend of ASMR and ASDR of atrial fibrillation in patients aged 30-45 years from 1990 to 2021 at global and socio-demographic index (SDI) levels by sex.

**Figure S3.** AAPC of ASMR and ASDR of atrial fibrillation in patients aged 30-45 years from 1990 to 2021 at SDI levels by sex.

**Figure S4.** AAPC of age-standardized prevalence, ASMR and ASDR of atrial fibrillation in patients aged 30-45 years from 1990 to 2021 by sex and age.

**Figure S5.** Prevalence (A), mortality (B) and DALYs (C) rate of atrial fibrillation in patients aged 30-45 years from 204 countries according to the SDI in 2021.

**Figure S6.** AAPC of age-standardized prevalence, ASMR and ASDR of atrial fibrillation in patients aged 30-45 years from 1990 to 2021 at regional levels.

**Figure S7.** AAPC of ASMR and ASDR of atrial fibrillation in patients aged 30-45 years from 1990 to 2021 at regional levels by sex.

**Supplementary Methods**

1. **Data sources**

GBD 2021 synthesises a large and growing number of data input sources including surveys, censuses, vital statistics, and other health-related data sources. The data from these sources are used to estimate morbidity; illness, and injury; and attributable risk for 204 countries and territories from 1990 to 2021; mortality deaths are estimated from 1980 to 2021.

The GBD estimation process is based on identifying multiple relevant data sources for each disease or injury, including censuses, household surveys, civil registration and vital statistics, disease registries, health service use, air pollution monitors, satellite imaging, disease notifications, and other sources. Each of these types of data is identified from a systematic review of published studies, searches of government and international organization websites, published reports, primary data sources such as the Demographic and Health Surveys, and contributions of datasets by GBD collaborators.

All data used in this study were extracted from the Global Health Data Exchange (https://vizhub.healthdata.org/gbd-results/) including (1) global age- and sex- specific prevalence, mortality, DALYs numbers and crude rates (per 100,000 persons) from 1990 to 2021; (2) Regional age- and sex- specific incidence, mortality, DALYs numbers and crude rates from 1990 to 2021 by socio-demographic index (SDI) categories; (3) National age- and sex- specific incidence, mortality, DALYs numbers and crude rates from 1990 to 2021; (4) GBD world standard population in 2021.

1. **Fatal disease modeling**

In the GBD 2021, vital registry data were utilized to model deaths from atrial fibrillation. The age-standardized mortality rate for atrial fibrillation was calculated per 100,000 populations via the Cause of Death Ensemble Model (CODEm) [1]. Deaths that were coded as having unclear intermediate causes in vital registration systems, or for which atrial fibrillation was not specified, were recoded via a multicause methodology.

1. **Nonfatal burdens modeling**

To estimate the nonfatal burdens of atrial fibrillation, the prevalence, age-standardized prevalence, and ASDR of atrial fibrillation were generated via the DisMod-MR 2.1 (Disease Model-Bayesian Meta-regression) modeling tool [2]. This approach considers the factors of sex, region, time, and age group to provide a comprehensive disease parameter estimate. Leveraging geospatial modeling, DisMod-MR 2.1 is not only able to process existing epidemiological data but also estimates disease prevalence for data-scarce regions using data from well-studied areas as priors, which allowed us to generate comprehensive prevalence and morbidity estimates.

1. **Processing of missing data**

The GBD 2021 input data were modelled by using Spatiotemporal Gaussian process regression (ST-GPR) modelling to allow for smoothing over age, time, and location in locations that were missing complete datasets. The flowchart showing the analytic steps can be found elsewhere [3]. The approach is a stochastic modelling technique that is designed to detect signals amidst noisy data. It also serves as a powerful tool for interpolating non-linear trends [4, 5]. The Bayesian noise reduction algorithm was used to deal with zero counts and small number issues for rare causes.

1. **Data quantification**

Age-standardized rates (ASR) were calculated per 100,000 people. Equations used to calculate ASR:

ASR=$\frac{\sum_{i=1}^{A} a_{i}w_{i}}{\sum_{i=1}^{A} w_{i}}$

where a_i_ is the age specifi­c rate and w_i_ is the weight in the same age subgroup of the chosen reference standard population (in which i denotes the i^th^ age class) and A is the upper age limit.

The AAPC is a statistical measure that captures the overall trend of change in a variable across a defined time span. In this study, the AAPC was calculated with slope coefficients from a base join-point regression model, which spans from 1990 to 2021 [6].

Equations used to calculate AAPC:

AAPC=$\left\{ \exp\left( \frac{\sum w_{i}b_{i}}{\Sigma w_{i}} \right)-1 \right\}\times100$
b_i_ is the slope coefficient for the i^th^ segment with i indexing the segments in the desired range of years, and w_i_ is the length of each segment in the range of years.

**Reference:**

1. GBD 2021 Causes of Death Collaborators . Global burden of 288 causes of death and life expectancy decomposition in 204 countries and territories and 811 subnational locations, 1990-2021: a systematic analysis for the global burden of disease study 2021. Lancet (London, England). (2024) 403:2100–32. doi: 10.1016/s0140-6736(24)00367-2
2. Global, regional, and national burden of stroke and its risk factors, 1990-2019: a systematic analysis for the global burden of disease study 2019. Lancet Neurol. (2021) 20:795–820. doi: 10.1016/s1474-4422(21)00252-0
3. GBD 2019 Risk Factors Collaborators. Global burden of 87 risk factors in 204 countries and territories, 1990–2019: a systematic analysis for the Global Burden of Disease Study 2019. The Lancet. doi: 10.1016/S0140-6736(20)30752-2
4. Vasudevan S, Ramos F, Nettleton E, Durrant-Whyte H, Blair A. Gaussian Process modeling of large scale terrain. In: 2009 IEEE International Conference on Robotics and Automation. 2009: 1047–53.
5. Rasmussen CE, Williams CKI. Gaussian Processes for Machine Learning. Cambridge, Mass: The MIT Press, 2005.
6. Kim HJ, Fay MP, Feuer EJ, Midthune DN. Permutation tests for joinpoint regression with applications to cancer rates. Stat Med. (2000) 19:335–51.

Supplementary Table 1. Age-standardized mortality rates (ASMR) and average annual percentage change (AAPC) of atrial fibrillation among people aged 30 - 45 years at the global and regional levels from 1990 to 2021

|  | Mortality (95% UI) |  |  |  |  |  |
| --- | --- | --- | --- | --- | --- | --- |
|  | Cases in 1990 | Age standardised rate in 1990 (per 100 000) | Cases in 2021 | Age standardised rate in 2021 (per 100 000) | AAPC (95% CI) | ΔR (95% CI) |
| Global | 418.34 (357.69 to 493.85) | 0.04 (0.04 to 0.05) | 747.97 (661.43 to 830.87) | 0.05 (0.04 to 0.05) | 0.17 (0.08 to 0.26) | 0.01 (-0.02 to 0.04) |
| Sex: |  |  |  |  |  |  |
| Female | 175.49 (142.84 to 222.77) | 0.04 (0.03 to 0.05) | 301.95 (255.65 to 344.07) | 0.04 (0.03 to 0.04) | 0.03 (-0.06 to 0.12) | 0 (-0.02 to 0.02) |
| Male | 242.85 (201.22 to 280.13) | 0.05 (0.04 to 0.06) | 446.02 (394.57 to 504.84) | 0.05 (0.05 to 0.06) | 0.3 (0.14 to 0.47) | 0 (-0.03 to 0.03) |
| Age group (years): |  |  |  |  |  |  |
| 30-34 | 69.04 (57.42 to 81.98) | 0.02 (0.01 to 0.02) | 114.67 (98.91 to 131.1) | 0.02 (0.02 to 0.02) | 0.16 (-0.06 to 0.37) | 0 (-0.01 to 0.01) |
| 35-39 | 99.99 (85.54 to 118.5) | 0.03 (0.02 to 0.03) | 174.91 (154.58 to 194.25) | 0.03 (0.03 to 0.03) | 0.32 (0.24 to 0.39) | 0 (-0.02 to 0.02) |
| 40-45 | 249.32 (214.72 to 293.37) | 0.09 (0.07 to 0.1) | 458.39 (407.94 to 505.52) | 0.09 (0.08 to 0.1) | 0.14 (0.03 to 0.25) | 0 (-0.06 to 0.02) |
| SDI level: |  |  |  |  |  |  |
| High | 89.72 (85 to 94.44) | 0.04 (0.04 to 0.05) | 108.44 (101.67 to 114.95) | 0.04 (0.04 to 0.05) | 0.08 (-0.11 to 0.27) | 0 (-0.02 to 0.02) |
| High-middle | 64.92 (56.92 to 73.41) | 0.03 (0.03 to 0.03) | 78.88 (69.98 to 87.63) | 0.03 (0.02 to 0.03) | -0.47 (-0.74 to -0.19) | 0 (-0.02 to 0.02) |
| Middle | 153.98 (131.54 to 177.73) | 0.05 (0.04 to 0.06) | 284.87 (248.11 to 318.59) | 0.05 (0.05 to 0.06) | 0.12 (0.03 to 0.22) | 0 (-0.03 to 0.03) |
| Low-middle | 80.86 (57.01 to 111.96) | 0.04 (0.03 to 0.06) | 198.41 (164.57 to 232.27) | 0.05 (0.04 to 0.06) | 0.49 (0.37 to 0.61) | 0.01 (-0.02 to 0.04) |
| Low | 28.11 (15.58 to 40.27) | 0.04 (0.02 to 0.06) | 76.24 (50.6 to 102.17) | 0.04 (0.03 to 0.06) | 0.23 (0.08 to 0.37) | 0 (-0.02 to 0.02) |

AAPC=average annual percentage change; CI=confidence interval; SDI=sociodemographic index; UI=uncertainty interval; ΔR=Absolute change in age-standardized rate (per 100,000 population)

Supplementary Table 2. Age-standardized DALYs (ASDR) and AAPC of atrial fibrillation among people aged 30 - 45 years at the global and regional levels from 1990 to 2021

|  | DALYs (95% UI) |  |  |  |  |  |
| --- | --- | --- | --- | --- | --- | --- |
|  | Cases in 1990 | Age standardised rate in 1990 (per 100 000) | Cases in 2021 | Age standardised rate in 2021 (per 100 000) | AAPC (95% CI) | ΔR (95% CI) |
| Global | 68557.93 (44152.71 to 107528.7) | 6.98 (4.49 to 10.94) | 117346.17 (78306.51 to 179783.58) | 7.08 (4.72 to 10.85) | 0.05 (-0.02 to 0.11) | 0.01 (-0.20 to 0.40) |
| Sex: |  |  |  |  |  |  |
| Female | 42930.1 (27416.17 to 66920.13) | 8.58 (5.48 to 13.38) | 72304.16 (48286.55 to 109363.57) | 8.65 (5.77 to 13.08) | 0.02 (-0.05 to 0.09) | 0.07 (-0.17 to 0.31) |
| Male | 25627.83 (16090.09 to 40043.72) | 5.31 (3.34 to 8.29) | 45042 (29563.86 to 69574.27) | 5.48 (3.6 to 8.47) | 0.11 (0.05 to 0.17) | 0.17 (-0.10 to 0.44) |
| Age group (years): |  |  |  |  |  |  |
| 30-34 | 6239.55 (4699.63 to 8429.46) | 1.62 (1.22 to 2.19) | 10077.92 (7925.49 to 13318.75) | 1.67 (1.31 to 2.2) | 0.09 (-0.04 to 0.22) | 0.05 (-0.03 to 0.13) |
| 35-39 | 18993.97 (11450.8 to 31608.26) | 5.39 (3.25 to 8.97) | 30885.21 (19366.64 to 49681.81) | 5.51 (3.45 to 8.86) | 0.08 (0.02 to 0.14) | 0.12 (-0.11 to 0.35) |
| 40-45 | 43324.41 (28002.28 to 67490.98) | 15.12 (9.77 to 23.56) | 76383.05 (51014.38 to 116783.02) | 15.27 (10.2 to 23.34) | 0.03 (-0.02 to 0.08) | 0.15 (-0.30 to 0.60) |
| SDI level: |  |  |  |  |  |  |
| High | 19659.9 (12351.13 to 31011.49) | 9.55 (6 to 15.06) | 24201.84 (16572.42 to 35158.6) | 9.95 (6.82 to 14.46) | 0.13 (0.04 to 0.22) | 0.40 (-0.19 to 0.99) |
| High-middle | 13917.95 (8348.8 to 22525.51) | 6.43 (3.87 to 10.38) | 20230.96 (12199.08 to 32973.41) | 6.66 (4.02 to 10.86) | 0.13 (0.01 to 0.24) | 0.23 (-0.12 to 0.58) |
| Middle | 21420.98 (14158.13 to 32858.33) | 6.98 (4.61 to 10.71) | 40288.43 (26628.21 to 62264.37) | 7.37 (4.87 to 11.4) | 0.18 (0.08 to 0.28) | 0.39 (-0.14 to 0.92) |
| Low-middle | 10043.28 (6351.61 to 15401.69) | 5.47 (3.46 to 8.4) | 23207.47 (15757.17 to 35382.96) | 5.99 (4.06 to 9.13) | 0.3 (0.18 to 0.42) | 0.52 (-0.10 to 1.14) |
| Low | 3430.96 (2058.33 to 5340.48) | 4.9 (2.94 to 7.62) | 9291.24 (5706.91 to 14610.28) | 5.24 (3.22 to 8.25) | 0.21 (0.14 to 0.28) | 0.34 (-0.10 to 0.78) |

AAPC=average annual percentage change; CI=confidence interval; SDI=sociodemographic index; UI=uncertainty interval；DALYs=disability-adjusted life years; ΔR=Absolute change in age-standardized rate (per 100,000 population)

Supplementary Table 3. Age-standardized prevalence of atrial fibrillation and its AAPC among adults aged 30–45 years at regional levels, 1990–2021.

|  | Prevalence (95% UI) |  |  |  |  |  |
| --- | --- | --- | --- | --- | --- | --- |
| Regions | Cases in 1990 | Age standardised rate in 1990 (per 100 000) | Cases in 2021 | Age standardised rate in 2021 (per 100 000) | AAPC (95% CI) | ΔR (95% CI) |
| Oceania | 577.42  (301.51 to 980.06) | 56.39 (29.56 to 95.56) | 1502.06  (798.57 to 2530.28) | 57.35 (30.53 to 96.56) | 0.06 (0.04 to 0.08) | 0.96 (-3.21 to 5.13) |
| Western Europe | 77506.93  (45162.51 to 121868.86) | 91.28 (53.07 to 143.7) | 93812.37  (62343.47 to 134204.22) | 102.62 (68 to 147.04) | 0.41 (0.26 to 0.56) | 11.34 (-2.07 to 24.75) |
| South Asia | 112169.22  (53958.57 to 202058.94) | 30.98 (14.97 to 55.68) | 248843.12  (122650.74 to 448984.33) | 31.98 (15.8 to 57.61) | 0.1 (0.08 to 0.12) | 1.00 (-4.88 to 6.88) |
| Central Latin America | 15869.5  (8482.73 to 26946.33) | 61.5 (33.05 to 104.17) | 34718.56  (18668.94 to 58622.11) | 63.11 (33.9 to 106.61) | 0.08 (0.07 to 0.1) | 1.61 (-5.27 to 8.49) |
| Andean Latin America | 3415.53  (1779.02 to 5808.77) | 56.68 (29.66 to 96.23) | 8697.47  (4666.8 to 14688.23) | 62.09 (33.33 to 104.85) | 0.29 (0.26 to 0.31) | 5.41 (-3.33 to 14.15) |
| East Asia | 134105.74  (69346.42 to 227667.62) | 54.93 (28.58 to 92.94) | 218436.2  (118548.54 to 362776.55) | 68.4 (37.18 to 113.51) | 0.72 (0.66 to 0.78) | 13.47 (-0.40 to 27.34) |
| Caribbean | 3954.12  (2144.38 to 6654.29) | 63.67 (34.61 to 107.04) | 6191.34  (3387.11 to 10379.31) | 64.32 (35.23 to 107.78) | 0.03 (0.02 to 0.04) | 0.65 (-6.81 to 8.11) |
| North Africa and Middle East | 31251.37  (15188.24 to 55062.03) | 30.9 (15.1 to 54.31) | 99609.69  (54169.57 to 165488.96) | 34.07 (18.54 to 56.57) | 0.31 (0.27 to 0.35) | 3.17 (-3.56 to 9.90) |
| Central Sub-Saharan Africa | 2507.41  (1243.34 to 4352) | 35.7 (17.92 to 61.57) | 7493.88  (3794.41 to 13005.05) | 35.81 (18.22 to 61.98) | 0.01 (-0.02 to 0.03) | 0.11 (-6.65 to 6.87) |
| Tropical Latin America | 26116.72  (14685.25 to 42592.55) | 96.26 (54.33 to 156.69) | 53692.3  (30343.17 to 87475.69) | 97.19 (54.84 to 158.44) | 0.22 (0.06 to 0.38) | 0.93 (-9.63 to 11.22) |
| Western Sub-Saharan Africa | 8612.49  (4219.21 to 15056.28) | 32.81 (16.17 to 57.16) | 25529.62  (12750.99 to 44675.72) | 35.51 (17.82 to 61.97) | 0.25 (0.24 to 0.27) | 2.70 (-4.35 to 9.75) |
| Central Asia | 4293.36  (2218.23 to 7390.69) | 43.48 (22.84 to 74.24) | 9247.55  (4764.51 to 15798.79) | 45.19 (23.37 to 77.1) | 0.11 (0.08 to 0.14) | 1.71 (-5.47 to 8.89) |
| Southern Sub-Saharan Africa | 3095.49  (1580.19 to 5436.5) | 38.75 (19.92 to 67.8) | 6764.84  (3428.59 to 11779.23) | 39.14 (19.94 to 67.96) | 0.01 (-0.01 to 0.02) | 0.39 (-7.88 to 8.66) |
| High-income Asia Pacific | 40082.89  (22637.88 to 65041.07) | 93.77 (52.73 to 152.42) | 44273.24  (29252.28 to 63171.35) | 110.24 (72.61 to 157.54) | 0.59 (0.2 to 0.99) | 16.47 (1.88 to 31.06) |
| Eastern Sub-Saharan Africa | 9656.61  (4858.62 to 16928.95) | 40.15 (20.37 to 70.07) | 29295.52  (14740.27 to 51038.15) | 44.5 (22.49 to 77.35) | 0.33 (0.29 to 0.38) | 4.35 (-4.88 to 13.58) |
| Southeast Asia | 45322.72  (24216.4 to 76823.67) | 59.78 (32.17 to 101) | 100339.62  (54069.44 to 170578.17) | 62.75 (33.76 to 106.76) | 0.15 (0.15 to 0.16) | 2.97 (-5.41 to 11.35) |
| Australasia | 6949.03  (5869.51 to 8268.35) | 144.81 (122.26 to 172.36) | 7315.85  (4218.23 to 11541.16) | 111.54 (64.3 to 175.96) | -0.95 (-1.09 to -0.81) | -33.27 (-66.06 to -0.48) |
| Eastern Europe | 30800.06  (16729.77 to 51578.17) | 64.44 (35.18 to 107.66) | 36120.02  (19958.4 to 59904.14) | 70.3 (38.84 to 116.6) | 0.28 (0.26 to 0.31) | 5.86 (-3.34 to 15.06) |
| Central Europe | 15313.69  (8252.61 to 25401.51) | 54.01 (29.14 to 89.54) | 17251.89  (10638.79 to 26439.3) | 61.23 (37.53 to 94.1) | 0.41 (0.33 to 0.49) | 7.22 (-0.61 to 15.05) |
| Southern Latin America | 4571.51  (2274.43 to 7841.04) | 47.72 (23.75 to 81.84) | 7738.31  (5152.54 to 11177.32) | 51.47 (34.24 to 74.39) | 0.25 (0.21 to 0.28) | 3.75 (-3.51 to 11.01) |
| High-income North America | 60945.84  (34259.24 to 98081.18) | 91.29 (51.34 to 146.88) | 69651  (54265.43 to 89041.62) | 91.69 (71.38 to 117.27) | 0.01 (-0.05 to 0.07) | 0.40 (-14.50 to 15.30) |

AAPC=average annual percentage change; CI=confidence interval; UI=uncertainty interval; ΔR=Absolute change in age-standardized rate (per 100,000 population)

Supplementary Table4. Age-standardized prevalence, ASMR and ASDR of atrial fibrillation among adults aged 30–45 years, by sex and region, 2021.

| Regions | Age-standardized rate in 2021 (per 100,000) | | |  |  |  |
| --- | --- | --- | --- | --- | --- | --- |
|  | Male |  |  | Female |  |  |
|  | Prevalence | Mortality | DALYs | Prevalence | Mortality | DALYs |
| Oceania | 62.63 (33.92 to 105.5) | 0.33 (0.18 to 0.5) | 21.75 (13.13 to 32.5) | 52.04 (26.93 to 88.34) | 0.38 (0.22 to 0.61) | 23.97 (14.88 to 36.49) |
| Western Europe | 174.36 (118.69 to 245.7) | 0.05 (0.05 to 0.05) | 17.21 (10.81 to 26.73) | 30.88 (16.82 to 49.52) | 0.02 (0.02 to 0.02) | 3.53 (2.17 to 5.6) |
| South Asia | 34.08 (17.02 to 60.66) | 0.05 (0.04 to 0.06) | 5.46 (3.63 to 8.18) | 29.81 (14.29 to 54.39) | 0.03 (0.02 to 0.04) | 3.85 (2.2 to 6.23) |
| Central Latin America | 74.04 (40.59 to 123.09) | 0.07 (0.06 to 0.08) | 9.64 (6.26 to 14.9) | 53.03 (27.7 to 89.92) | 0.06 (0.05 to 0.07) | 7.37 (4.96 to 11.14) |
| Andean Latin America | 73.67 (39.97 to 125.4) | 0.07 (0.05 to 0.1) | 9.81 (5.87 to 16.12) | 50.95 (26.71 to 86.69) | 0.06 (0.04 to 0.09) | 7.31 (4.31 to 11.6) |
| East Asia | 73.74 (40.6 to 121.65) | 0.02 (0.01 to 0.03) | 7.23 (4.01 to 12.51) | 62.77 (33.49 to 105.27) | 0.01 (0.01 to 0.02) | 5.84 (2.96 to 10.17) |
| Caribbean | 74.67 (41.58 to 123.61) | 0.09 (0.07 to 0.12) | 11.01 (7.25 to 17.06) | 54.31 (28.54 to 92.32) | 0.07 (0.05 to 0.09) | 7.95 (5.08 to 12.31) |
| North Africa and Middle East | 43.57 (23.78 to 71.43) | 0.04 (0.03 to 0.05) | 5.57 (3.58 to 8.66) | 23.29 (11.2 to 40.22) | 0.05 (0.04 to 0.06) | 4.33 (3.04 to 6.23) |
| Central Sub-Saharan Africa | 38.06 (19.57 to 65.48) | 0.07 (0.04 to 0.11) | 6.48 (3.49 to 10.58) | 33.59 (17.34 to 58.96) | 0.03 (0.02 to 0.06) | 4.45 (2.01 to 8.24) |
| Tropical Latin America | 118.05 (68.39 to 190.95) | 0.1 (0.09 to 0.1) | 14.76 (9.79 to 23.21) | 77.32 (42.2 to 127.56) | 0.07 (0.07 to 0.08) | 9.79 (6.38 to 15.15) |
| Western Sub-Saharan Africa | 37.99 (19.29 to 66) | 0.02 (0.01 to 0.03) | 4.45 (2.5 to 7.59) | 33.23 (16.36 to 58.19) | 0.01 (0.01 to 0.02) | 3.36 (1.76 to 5.81) |
| Central Asia | 70.33 (37.98 to 116.49) | 0.04 (0.03 to 0.05) | 7.98 (4.62 to 13.4) | 20.44 (8.46 to 37.07) | 0.01 (0.01 to 0.01) | 2.22 (1.02 to 4.17) |
| Southern Sub-Saharan Africa | 41.91 (21.43 to 72.49) | 0.11 (0.08 to 0.15) | 9.06 (6.45 to 12.49) | 36.38 (18.5 to 63.43) | 0.13 (0.09 to 0.17) | 9.46 (6.71 to 13.19) |
| High-income Asia Pacific | 136.06 (89.84 to 193.87) | 0.05 (0.04 to 0.06) | 13.91 (8.43 to 21.46) | 83.05 (55.09 to 115.62) | 0.02 (0.02 to 0.03) | 8.06 (4.68 to 12.58) |
| Eastern Sub-Saharan Africa | 45.72 (23.13 to 79.29) | 0.07 (0.04 to 0.12) | 7.48 (4.4 to 11.89) | 43.36 (22.19 to 75.51) | 0.03 (0.02 to 0.05) | 5 (2.85 to 8.18) |
| Southeast Asia | 67.3 (36.61 to 114.79) | 0.1 (0.08 to 0.13) | 11.12 (7.59 to 16.35) | 58.13 (30.77 to 98.5) | 0.1 (0.08 to 0.13) | 10 (7 to 14.31) |
| Australasia | 168.23 (99.07 to 262.61) | 0.06 (0.05 to 0.07) | 16.88 (9.34 to 29.38) | 56.15 (30.38 to 93.1) | 0.02 (0.02 to 0.02) | 5.67 (2.22 to 11.18) |
| Eastern Europe | 111.6 (63.23 to 179.56) | 0.04 (0.03 to 0.04) | 11.12 (6.43 to 18.38) | 30.53 (14.67 to 56.07) | 0.01 (0.01 to 0.01) | 3.19 (1.71 to 5.7) |
| Central Europe | 101.16 (63.22 to 151.87) | 0.03 (0.03 to 0.04) | 10.19 (6.24 to 16.53) | 19.75 (9.83 to 33.04) | 0.01 (0.01 to 0.01) | 2.2 (1.24 to 3.65) |
| Southern Latin America | 81.22 (56.03 to 114.8) | 0.05 (0.04 to 0.06) | 9.38 (5.62 to 14.81) | 22.76 (12.67 to 36.24) | 0.03 (0.03 to 0.04) | 3.65 (2.29 to 5.59) |
| High-income North America | 135.59 (107.5 to 172.23) | 0.09 (0.09 to 0.1) | 15.95 (11.98 to 21.08) | 48.6 (36.3 to 64.21) | 0.04 (0.04 to 0.04) | 5.97 (4.46 to 8.03) |

DALYs=Disability-Adjusted Life Years; ASMR=Age-standardized mortality rates; ASDR= Age-standardized DALYs

Supplementary Table 5. ASMR of atrial fibrillation and its AAPC among adults aged 30–45 years at regional levels, 1990–2021.

|  | Mortality (95% UI) | |  |  |  |  |
| --- | --- | --- | --- | --- | --- | --- |
| Regions | Cases in 1990 | Age standardised rate in 1990 (per 100 000) | Cases in 2021 | Age standardised rate in 2021 (per 100 000) | AAPC (95% CI) | ΔR (95% CI) |
| Oceania | 3.62  (2.06 to 5.4) | 0.34 (0.19 to 0.5) | 9.39  (5.85 to 13.66) | 0.35 (0.22 to 0.51) | 0.14 (-0.1 to 0.38) | 0.01 (-0.13 to 0.15) |
| Western Europe | 41.09  (38.64 to 43.52) | 0.05 (0.05 to 0.05) | 31.87  (30.05 to 33.81) | 0.04 (0.03 to 0.04) | -1.09 (-1.53 to -0.66) | -0.01 (-0.02 to 0) |
| South Asia | 117.85  (69.84 to 175.73) | 0.03 (0.02 to 0.05) | 312.58  (239.24 to 383.45) | 0.04 (0.03 to 0.05) | 0.68 (0.64 to 0.72) | 0.01 (-0.02 to 0.04) |
| Central Latin America | 17.45  (16.63 to 18.34) | 0.07 (0.06 to 0.07) | 35.67  (31.63 to 39.96) | 0.06 (0.06 to 0.07) | -0.16 (-0.5 to 0.18) | -0.01 (-0.01 to 0) |
| Andean Latin America | 6  (4.54 to 7.72) | 0.1 (0.07 to 0.12) | 9.5  (7.23 to 12.47) | 0.07 (0.05 to 0.09) | -1.11 (-1.45 to -0.77) | -0.03 (-0.07 to 0.01) |
| East Asia | 51.07  (39.07 to 63.77) | 0.02 (0.02 to 0.03) | 49.53  (37.64 to 62.65) | 0.02 (0.01 to 0.02) | -1.05 (-1.34 to -0.77) | 0 (-0.01 to 0.01) |
| Caribbean | 5.14  (4.37 to 6.02) | 0.08 (0.07 to 0.1) | 7.96  (6.4 to 9.83) | 0.08 (0.07 to 0.1) | 0.03 (-0.29 to 0.35) | 0 (-0.03 to 0.03) |
| North Africa and Middle East | 51.4  (39.22 to 69.25) | 0.05 (0.04 to 0.07) | 125.17  (104.37 to 152.57) | 0.04 (0.04 to 0.05) | -0.46 (-0.55 to -0.36) | -0.01 (-0.03 to 0.01) |
| Central Sub-Saharan Africa | 3.43  (1.84 to 5.51) | 0.05 (0.03 to 0.08) | 10.73  (6.48 to 16.66) | 0.05 (0.03 to 0.08) | 0.16 (0.08 to 0.24) | 0 (-0.05 to 0.05) |
| Tropical Latin America | 20.3  (19.34 to 21.46) | 0.07 (0.07 to 0.08) | 45.68  (43.52 to 48.13) | 0.08 (0.08 to 0.09) | 0.3 (-0.34 to 0.95) | 0.01 (0 to 0.02) |
| Western Sub-Saharan Africa | 5  (3.57 to 6.58) | 0.02 (0.01 to 0.02) | 13.35  (8.76 to 17.92) | 0.02 (0.01 to 0.02) | -0.06 (-0.16 to 0.05) | 0 (-0.01 to 0.01) |
| Central Asia | 2.77  (2.49 to 3.13) | 0.03 (0.02 to 0.03) | 5.1  (4.41 to 5.9) | 0.02 (0.02 to 0.03) | -0.21 (-0.6 to 0.18) | -0.01 (-0.01 to 0) |
| Southern Sub-Saharan Africa | 11.26  (9.21 to 13.48) | 0.13 (0.11 to 0.16) | 21.22  (16.89 to 26.86) | 0.12 (0.1 to 0.15) | -0.35 (-1.31 to 0.61) | -0.01 (-0.06 to 0.04) |
| High-income Asia Pacific | 22.13  (19.16 to 25.47) | 0.05 (0.05 to 0.06) | 14.23  (12.79 to 16.3) | 0.04 (0.03 to 0.04) | -1.18 (-1.61 to -0.75) | -0.01 (-0.02 to 0) |
| Eastern Sub-Saharan Africa | 13.48  (7.36 to 19.31) | 0.05 (0.03 to 0.08) | 33.59  (20.29 to 51.44) | 0.05 (0.03 to 0.08) | -0.28 (-0.37 to -0.19) | 0 (-0.05 to 0.05) |
| Southeast Asia | 80.65  (60.67 to 99.14) | 0.1 (0.08 to 0.12) | 162.73  (130.45 to 195.07) | 0.1 (0.08 to 0.12) | 0.13 (-0.01 to 0.26) | 0 (-0.04 to 0.04) |
| Australasia | 1.74  (1.56 to 1.94) | 0.04 (0.03 to 0.04) | 2.49  (2.18 to 2.82) | 0.04 (0.03 to 0.04) | 0.2 (-0.46 to 0.87) | 0 (-0.01 to 0.01) |
| Eastern Europe | 7.94  (6.33 to 9.89) | 0.02 (0.01 to 0.02) | 12.59  (11.49 to 13.68) | 0.02 (0.02 to 0.03) | 1.37 (0.92 to 1.83) | 0 (-0.01 to 0.01) |
| Central Europe | 10.1  (9.48 to 10.84) | 0.04 (0.03 to 0.04) | 6.17  (5.53 to 6.86) | 0.02 (0.02 to 0.02) | -1.71 (-1.97 to -1.45) | -0.02 (-0.02 to -0.01) |
| Southern Latin America | 4.82  (4.34 to 5.35) | 0.05 (0.05 to 0.06) | 6.23  (5.6 to 6.94) | 0.04 (0.04 to 0.05) | -0.57 (-0.96 to -0.17) | -0.01 (-0.02 to 0) |
| High-income North America | 25.75  (24.87 to 26.72) | 0.04 (0.04 to 0.04) | 51.06  (47.7 to 54.16) | 0.07 (0.06 to 0.07) | 1.82 (1.55 to 2.08) | 0.03 (0.02 to 0.03) |

AAPC=average annual percentage change; CI=confidence interval; UI=uncertainty interval; ASMR=Age-standardized mortality rates; ΔR=Absolute change in age-standardized rate (per 100,000 population)

Supplementary Table 6. ASDR of atrial fibrillation and its AAPC among adults aged 30–45 years at regional levels, 1990–2021.

|  | DALYs (95% UI) |  |  |  |  |  |
| --- | --- | --- | --- | --- | --- | --- |
| Regions | Cases in 1990 | Age standardised rate in 1990 (per 100 000) | Cases in 2021 | Age standardised rate in 2021 (per 100 000) | AAPC (95% CI) | ΔR (95% CI) |
| Oceania | 234.6  (146.75 to 338.04) | 22.05 (13.86 to 31.66) | 608.89  (401.82 to 871.84) | 22.88 (15.11 to 32.74) | 0.12 (-0.08 to 0.32) | 0.83 (-8.80 to 10.88) |
| Western Europe | 8572.15  (5326.45 to 13361.9) | 10.12 (6.29 to 15.78) | 9448.84  (6041.85 to 14430.54) | 10.37 (6.63 to 15.84) | 0.11 (-0.02 to 0.24) | 0.25 (-9.15 to 9.55) |
| South Asia | 15357.06  (9178.66 to 24327.78) | 4.21 (2.52 to 6.67) | 36436.46  (23394.68 to 57076.73) | 4.67 (3 to 7.31) | 0.33 (0.31 to 0.35) | 0.46 (-3.67 to 4.79) |
| Central Latin America | 2196.63  (1491.77 to 3296.83) | 8.41 (5.7 to 12.65) | 4646.25  (3084.28 to 7057.14) | 8.46 (5.61 to 12.84) | 0.01 (-0.14 to 0.16) | 0.05 (-7.04 to 7.43) |
| Andean Latin America | 589.92  (405.61 to 859.42) | 9.62 (6.61 to 14.06) | 1197.9  (747.51 to 1877.33) | 8.54 (5.32 to 13.38) | -0.35 (-0.51 to -0.19) | -1.08 (-8.74 to 4.77) |
| East Asia | 13850.35  (7739.33 to 23289.64) | 5.7 (3.22 to 9.55) | 20941.82  (11308.85 to 35824.44) | 6.56 (3.54 to 11.21) | 0.45 (0.41 to 0.5) | 0.86 (-6.01 to 7.99) |
| Caribbean | 590.61 (403.53 to 874.65) | 9.43 (6.43 to 13.98) | 913.96  (617.65 to 1363.74) | 9.46 (6.39 to 14.12) | 0 (-0.17 to 0.17) | 0.03 (-7.59 to 7.69) |
| North Africa and Middle East | 5233.29  (3468.92 to 7776.79) | 5.11 (3.38 to 7.58) | 14555.36  (9995.56 to 21567.6) | 4.99 (3.43 to 7.38) | -0.08 (-0.16 to 0.01) | -0.12 (-4.15 to 4.00) |
| Central Sub-Saharan Africa | 377.32  (208.6 to 604.29) | 5.31 (2.97 to 8.46) | 1152.69  (646.35 to 1913.21) | 5.46 (3.06 to 9.07) | 0.12 (0.01 to 0.22) | 0.15 (-5.40 to 6.10) |
| Tropical Latin America | 3190.78  (2093.76 to 4941.24) | 11.68 (7.65 to 18.09) | 6722.01  (4544.21 to 10376.67) | 12.22 (8.27 to 18.84) | 0.26 (0.01 to 0.51) | 0.54 (-9.82 to 11.19) |
| Western Sub-Saharan Africa | 982.83  (559.55 to 1626.18) | 3.67 (2.08 to 6.1) | 2828.76  (1594.46 to 4837.4) | 3.88 (2.18 to 6.65) | 0.17 (0.13 to 0.2) | 0.21 (-4.02 to 4.57) |
| Central Asia | 504.98  (298.26 to 854.26) | 5.02 (2.97 to 8.47) | 1043.67  (595.61 to 1748.03) | 5.08 (2.9 to 8.51) | 0.05 (-0.08 to 0.17) | 0.06 (-5.57 to 5.54) |
| Southern Sub-Saharan Africa | 840.38  (647.1 to 1116.62) | 10.02 (7.7 to 13.41) | 1632.36  (1236.73 to 2223.22) | 9.27 (7.02 to 12.64) | -0.27 (-0.95 to 0.41) | -0.75 (-6.39 to 4.62) |
| High-income Asia Pacific | 4469.04  (2787.97 to 7081.5) | 10.52 (6.55 to 16.66) | 4422.86  (2723.82 to 6704.23) | 11.06 (6.81 to 16.79) | 0.18 (-0.13 to 0.48) | 0.54 (-9.85 to 10.24) |
| Eastern Sub-Saharan Africa | 1478.49  (891.47 to 2261.27) | 6.04 (3.66 to 9.24) | 4128.7  (2439.29 to 6588.9) | 6.2 (3.67 to 9.91) | 0.09 (0.03 to 0.15) | 0.16 (-5.57 to 6.25) |
| Southeast Asia | 7991.63  (5490.35 to 11510.72) | 10.15 (6.95 to 14.72) | 16780.43  (11956.15 to 23969.47) | 10.57 (7.54 to 15.08) | 0.14 (0.08 to 0.2) | 0.42 (-7.77 to 8.13) |
| Australasia | 662.58  (420.13 to 978.06) | 13.8 (8.73 to 20.42) | 734.96  (406.52 to 1276.57) | 11.21 (6.2 to 19.47) | -0.79 (-0.86 to -0.72) | -2.59 (-14.22 to 7.67) |
| Eastern Europe | 2972.6  (1662.46 to 4990.94) | 6.22 (3.49 to 10.42) | 3632.05  (2120.26 to 6041.68) | 7.08 (4.14 to 11.77) | 0.42 (0.33 to 0.52) | 0.86 (-7.28 to 8.28) |
| Central Europe | 1787.85  (1139.05 to 2834.87) | 6.31 (4.02 to 10) | 1761.7  (1079.53 to 2851.03) | 6.27 (3.83 to 10.16) | -0.03 (-0.19 to 0.13) | -0.04 (-6.17 to 6.14) |
| Southern Latin America | 625.64  (389.72 to 1001.13) | 6.52 (4.06 to 10.44) | 969.97  (615.25 to 1456.2) | 6.46 (4.1 to 9.7) | -0.04 (-0.13 to 0.06) | -0.06 (-6.34 to 5.64) |
| High-income North America | 6344.38  (3844.65 to 10365.88) | 9.5 (5.76 to 15.52) | 8282.41  (6270.83 to 10879.53) | 10.91 (8.26 to 14.35) | 0.45 (0.34 to 0.56) | 1.41 (-7.26 to 8.59) |

AAPC=average annual percentage change; CI=confidence interval; UI=uncertainty interval; DALYs=Disability-Adjusted Life Years; ASDR= Age-standardized DALYs; ΔR=Absolute change in age-standardized rate (per 100,000 population)

Supplementary Table7. Age-standardized prevalence, ASMR and ASDR of atrial fibrillation in adults aged 30–45 years in 2021 and their AAPCs between 1990-2021 in 204 countries and territories.

|  | Age-standardized rate in 2021 (per 100,000) | | | AAPC 1990-20021 (95% CI) | | |
| --- | --- | --- | --- | --- | --- | --- |
|  | Prevalence | Mortality | DALYs | Prevalence | Mortality | DALYs |
| Afghanistan | 30.84 (14.99 to 53.02) | 0.05 (0.03 to 0.1) | 5.11 (2.57 to 8.52) | 0.44 (0.38 to 0.51) | 0.15 (-0.11 to 0.41) | 0.29 (0.07 to 0.52) |
| Albania | 51.98 (27.64 to 88.91) | 0.02 (0.01 to 0.03) | 5.47 (2.52 to 10.25) | -0.11 (-0.13 to -0.09) | -0.06 (-1.19 to 1.07) | -0.08 (-0.23 to 0.08) |
| Algeria | 33.04 (15.99 to 57.5) | 0.04 (0.02 to 0.07) | 4.69 (2.41 to 8.14) | 0.19 (0.12 to 0.27) | -0.22 (-0.37 to -0.07) | -0.01 (-0.12 to 0.1) |
| American Samoa | 67.86 (36.36 to 113.2) | 0.45 (0.28 to 0.66) | 28.66 (18.63 to 40.77) | 0.28 (0.25 to 0.31) | 0.75 (0.62 to 0.88) | 0.65 (0.54 to 0.76) |
| Andorra | 84.05 (48.76 to 137.98) | 0.02 (0.01 to 0.03) | 8.13 (3.92 to 14.4) | -0.46 (-0.54 to -0.37) | -1.48 (-1.97 to -0.98) | -0.63 (-0.86 to -0.4) |
| Angola | 37.59 (19.23 to 66.74) | 0.05 (0.03 to 0.09) | 5.81 (3.09 to 9.78) | 0.21 (0.19 to 0.23) | 0.13 (-0.4 to 0.66) | 0.15 (0.03 to 0.27) |
| Antigua and Barbuda | 62.88 (33.48 to 105.53) | 0.04 (0.04 to 0.05) | 7.37 (4 to 12.55) | 0.09 (0.05 to 0.12) | -1.82 (-2.66 to -0.97) | -0.79 (-1.34 to -0.23) |
| Argentina | 51.38 (35.87 to 70.59) | 0.04 (0.03 to 0.05) | 6.32 (3.85 to 10.14) | 0.33 (0.29 to 0.36) | -0.87 (-1.66 to -0.08) | -0.05 (-0.32 to 0.21) |
| Armenia | 46.27 (24.27 to 81.1) | 0.02 (0.02 to 0.02) | 4.92 (2.22 to 9.07) | 0.28 (0.22 to 0.33) | 0.85 (-0.19 to 1.9) | 0.26 (0.06 to 0.45) |
| Australia | 113.56 (64.33 to 181.57) | 0.04 (0.03 to 0.04) | 11.26 (6.04 to 19.89) | -1.16 (-1.29 to -1.02) | 0.24 (-0.45 to 0.93) | -0.99 (-1.11 to -0.87) |
| Austria | 115.39 (99.27 to 136.27) | 0.04 (0.03 to 0.05) | 11.73 (7.43 to 17.59) | 1.3 (1.25 to 1.34) | -1.44 (-2.03 to -0.83) | 0.64 (0.49 to 0.8) |
| Azerbaijan | 46.13 (24.24 to 78.93) | 0.02 (0.01 to 0.03) | 4.77 (2.14 to 9.26) | 0.29 (0.26 to 0.32) | -1.05 (-1.55 to -0.55) | 0.05 (-0.23 to 0.32) |
| Bahamas | 64.74 (34.57 to 109.28) | 0.12 (0.09 to 0.17) | 11.56 (7.84 to 17.45) | -0.07 (-0.1 to -0.05) | -0.1 (-0.36 to 0.17) | -0.08 (-0.24 to 0.07) |
| Bahrain | 37.93 (18.28 to 64.68) | 0.02 (0.01 to 0.04) | 4.01 (1.56 to 7.91) | 0.14 (-0.05 to 0.32) | -0.66 (-1.4 to 0.08) | -0.13 (-0.28 to 0.03) |
| Bangladesh | 31.83 (15.8 to 56) | 0.03 (0.02 to 0.06) | 4.38 (2.15 to 7.76) | 0.1 (0.07 to 0.13) | 0.12 (-0.41 to 0.66) | 0.11 (-0.11 to 0.33) |
| Barbados | 67.2 (36.24 to 112.71) | 0.05 (0.04 to 0.07) | 8.16 (4.52 to 13.44) | 0.15 (0.11 to 0.2) | -1.08 (-2.37 to 0.23) | -0.32 (-0.86 to 0.23) |
| Belarus | 78.87 (42.92 to 131.22) | 0.02 (0.01 to 0.02) | 7.45 (3.55 to 14.1) | 0.36 (0.32 to 0.4) | 0.77 (-0.07 to 1.61) | 0.38 (0.16 to 0.59) |
| Belgium | 145.55 (88.46 to 212.17) | 0.03 (0.03 to 0.04) | 13.97 (7.51 to 23.89) | 1.95 (1.83 to 2.07) | -0.79 (-1.1 to -0.49) | 1.47 (1.26 to 1.68) |
| Belize | 65.48 (35.15 to 111.42) | 0.07 (0.06 to 0.08) | 8.96 (5.34 to 14.01) | 0.1 (0.08 to 0.13) | 0.14 (-1.18 to 1.47) | 0.09 (-0.39 to 0.58) |
| Benin | 34.58 (17.25 to 59.87) | 0.02 (0.01 to 0.02) | 3.67 (1.43 to 7.32) | 0.14 (0.12 to 0.17) | 0.52 (0.38 to 0.66) | 0.2 (0.07 to 0.33) |
| Bermuda | 66.92 (35.91 to 110.22) | 0.03 (0.02 to 0.04) | 7.14 (3.5 to 12.96) | -0.05 (-0.06 to -0.03) | -2.6 (-3.1 to -2.1) | -0.65 (-0.74 to -0.56) |
| Bhutan | 32.15 (16.02 to 57.1) | 0.03 (0.02 to 0.06) | 4.4 (2.2 to 7.73) | 0.11 (0.09 to 0.14) | 0.79 (0.58 to 1) | 0.37 (0.14 to 0.6) |
| Bolivia (Plurinational State of) | 60.47 (31.92 to 101.82) | 0.08 (0.05 to 0.13) | 9.26 (5.34 to 14.96) | 0.2 (0.18 to 0.22) | -0.92 (-1.09 to -0.75) | -0.38 (-0.45 to -0.31) |
| Bosnia and Herzegovina | 52.94 (27.33 to 89.35) | 0.02 (0.01 to 0.03) | 5.51 (2.6 to 10) | 0.12 (0.06 to 0.18) | -1.23 (-1.9 to -0.57) | -0.2 (-0.51 to 0.11) |
| Botswana | 39.15 (19.59 to 69.54) | 0.07 (0.04 to 0.11) | 6.65 (3.75 to 11.06) | 0.19 (0.17 to 0.22) | -0.91 (-1.67 to -0.15) | -0.44 (-0.92 to 0.03) |
| Brazil | 96.74 (54.49 to 157.78) | 0.08 (0.08 to 0.09) | 12.2 (8.27 to 18.8) | 0.23 (0.07 to 0.39) | 0.43 (-0.28 to 1.14) | 0.27 (0.02 to 0.52) |
| Brunei Darussalam | 97.55 (56.39 to 154.39) | 0.12 (0.09 to 0.16) | 14.17 (8.99 to 21.3) | -0.57 (-0.59 to -0.54) | -0.22 (-0.59 to 0.15) | -0.4 (-0.56 to -0.25) |
| Bulgaria | 52.95 (28.37 to 90.79) | 0.04 (0.03 to 0.05) | 6.54 (3.75 to 11.39) | 0.08 (0.04 to 0.12) | -0.77 (-1.48 to -0.04) | -0.24 (-0.57 to 0.1) |
| Burkina Faso | 34.62 (17.31 to 58.66) | 0.02 (0.01 to 0.03) | 3.81 (1.51 to 7.64) | 0.16 (0.14 to 0.17) | 0.5 (0.23 to 0.77) | 0.31 (0.17 to 0.45) |
| Burundi | 42.8 (21.77 to 75.17) | 0.04 (0.02 to 0.07) | 5.54 (2.76 to 9.99) | 0.05 (0.05 to 0.06) | -1.1 (-1.22 to -0.98) | -0.43 (-0.54 to -0.33) |
| Cabo Verde | 36.57 (18.51 to 64.54) | 0.02 (0.01 to 0.03) | 4.01 (1.68 to 7.69) | 0.24 (0.23 to 0.26) | -0.85 (-1.34 to -0.36) | -0.13 (-0.21 to -0.04) |
| Cambodia | 58.96 (31.13 to 99.4) | 0.08 (0.05 to 0.12) | 8.85 (5.29 to 14.61) | 0.11 (0.07 to 0.16) | -0.23 (-0.31 to -0.14) | -0.02 (-0.07 to 0.03) |
| Cameroon | 37.29 (19.07 to 65.93) | 0.03 (0.01 to 0.04) | 4.46 (2 to 8.14) | 0.34 (0.33 to 0.36) | 0.46 (0.33 to 0.58) | 0.38 (0.3 to 0.46) |
| Canada | 79.21 (44.42 to 130.71) | 0.04 (0.04 to 0.05) | 8.73 (4.63 to 15.01) | -0.45 (-0.63 to -0.27) | 0.81 (0.39 to 1.24) | -0.21 (-0.4 to -0.03) |
| Central African Republic | 35.36 (17.99 to 60.25) | 0.06 (0.03 to 0.11) | 6.06 (2.97 to 10.64) | 0 (-0.02 to 0.02) | 0.24 (-0.08 to 0.56) | 0.15 (-0.07 to 0.37) |
| Chad | 33.23 (16.36 to 57.21) | 0.02 (0.01 to 0.03) | 3.67 (1.56 to 7) | 0.11 (0.1 to 0.12) | 1.23 (0.99 to 1.47) | 0.38 (0.34 to 0.42) |
| Chile | 52.4 (27.77 to 90.97) | 0.05 (0.04 to 0.06) | 6.86 (3.92 to 11.65) | 0.1 (0.07 to 0.13) | -0.39 (-0.95 to 0.18) | -0.02 (-0.32 to 0.28) |
| China | 68.18 (37.05 to 113.16) | 0.02 (0.01 to 0.02) | 6.52 (3.51 to 11.16) | 0.75 (0.69 to 0.81) | -1.13 (-1.43 to -0.83) | 0.49 (0.44 to 0.53) |
| Colombia | 62.62 (33.86 to 104.85) | 0.05 (0.04 to 0.06) | 7.58 (4.1 to 12.59) | 0.06 (0.04 to 0.08) | -1.03 (-1.61 to -0.43) | -0.4 (-0.48 to -0.32) |
| Comoros | 42.14 (21.23 to 72.56) | 0.05 (0.02 to 0.09) | 5.84 (2.82 to 10.62) | 0.11 (0.1 to 0.12) | -1.1 (-2.24 to 0.06) | -0.42 (-0.91 to 0.06) |
| Congo | 38.08 (19.16 to 64.15) | 0.07 (0.04 to 0.12) | 6.64 (3.41 to 10.89) | 0.14 (0.12 to 0.15) | -0.58 (-1.13 to -0.03) | -0.27 (-0.61 to 0.07) |
| Cook Islands | 67.76 (36.7 to 117.52) | 0.37 (0.22 to 0.59) | 24.78 (15.39 to 37.33) | 0.43 (0.38 to 0.47) | -0.68 (-0.78 to -0.57) | -0.48 (-0.61 to -0.34) |
| Costa Rica | 69.06 (36.77 to 116.31) | 0.05 (0.04 to 0.06) | 8.24 (4.64 to 13.53) | -0.02 (-0.03 to -0.01) | 0.45 (-0.03 to 0.94) | 0.09 (-0.07 to 0.25) |
| Coted'Ivoire | 36.36 (18.07 to 64.04) | 0.02 (0.01 to 0.03) | 4.17 (1.87 to 8.14) | 0.13 (0.07 to 0.18) | 0.7 (0.43 to 0.98) | 0.31 (0.05 to 0.57) |
| Croatia | 49.31 (32.59 to 68.66) | 0.01 (0.01 to 0.01) | 4.7 (2.26 to 8.62) | -0.31 (-0.33 to -0.28) | -0.64 (-2.76 to 1.52) | -0.36 (-0.59 to -0.13) |
| Cuba | 64.33 (35.51 to 108.31) | 0.05 (0.04 to 0.06) | 7.93 (4.4 to 13.16) | 0 (-0.01 to 0) | -0.85 (-1.33 to -0.36) | -0.32 (-0.57 to -0.08) |
| Cyprus | 51.33 (30.32 to 79.66) | 0.03 (0.02 to 0.04) | 5.73 (2.85 to 9.78) | -1.17 (-1.21 to -1.13) | -1.46 (-2.28 to -0.63) | -1.29 (-1.68 to -0.91) |
| Czechia | 82.76 (55.65 to 110) | 0.02 (0.01 to 0.02) | 7.93 (4.33 to 12.97) | 1.05 (0.94 to 1.17) | -1.42 (-2.21 to -0.62) | 0.71 (0.43 to 0.98) |
| Democratic People's Republic of Korea | 68.67 (37.12 to 113.8) | 0.03 (0.02 to 0.05) | 7.24 (3.53 to 12.59) | -0.01 (-0.04 to 0.02) | 0.36 (0.24 to 0.48) | 0.07 (-0.05 to 0.18) |
| Democratic Republic of the Congo | 34.91 (17.74 to 60.95) | 0.05 (0.02 to 0.08) | 5.18 (2.61 to 9.11) | -0.09 (-0.13 to -0.04) | 0.28 (0.15 to 0.4) | 0.09 (0.02 to 0.17) |
| Denmark | 139.12 (86.98 to 206.81) | 0.04 (0.03 to 0.05) | 13.6 (7.22 to 23.58) | 0.7 (0.61 to 0.79) | -0.83 (-1.19 to -0.47) | 0.45 (0.35 to 0.56) |
| Djibouti | 43.15 (21.81 to 75.62) | 0.05 (0.03 to 0.08) | 6.04 (3.08 to 10.24) | 0.23 (0.21 to 0.24) | -0.65 (-0.92 to -0.39) | -0.17 (-0.29 to -0.04) |
| Dominica | 65.04 (35.14 to 109.88) | 0.08 (0.05 to 0.12) | 9.49 (5.4 to 15.05) | 0.06 (0.04 to 0.07) | 0.43 (0.16 to 0.7) | 0.2 (0.05 to 0.35) |
| Dominican Republic | 64.23 (34.84 to 106.42) | 0.09 (0.07 to 0.13) | 10.14 (6.26 to 15.85) | 0.09 (0.06 to 0.11) | 0.26 (-0.28 to 0.79) | 0.11 (-0.13 to 0.36) |
| Ecuador | 59.28 (31.08 to 99.98) | 0.06 (0.05 to 0.08) | 8.04 (4.54 to 12.53) | 0.1 (0.08 to 0.13) | -1.66 (-2.76 to -0.55) | -0.65 (-1.28 to -0.01) |
| Egypt | 34.03 (16.48 to 58.63) | 0.05 (0.03 to 0.06) | 5.17 (2.88 to 8.42) | 0.41 (0.39 to 0.43) | -0.55 (-1.01 to -0.1) | -0.07 (-0.32 to 0.19) |
| El Salvador | 63.37 (34.34 to 104.97) | 0.1 (0.07 to 0.14) | 10.09 (6.17 to 15.66) | 0.08 (0.04 to 0.12) | -0.04 (-1.01 to 0.95) | 0.04 (-0.52 to 0.6) |
| Equatorial Guinea | 40.3 (20.42 to 69.04) | 0.06 (0.03 to 0.11) | 6.22 (3.1 to 10.49) | 0.49 (0.42 to 0.55) | -0.03 (-0.83 to 0.77) | 0.27 (0.15 to 0.39) |
| Eritrea | 40.41 (20.6 to 68.46) | 0.07 (0.04 to 0.12) | 6.86 (3.63 to 11.16) | 0.13 (0.12 to 0.15) | -0.67 (-0.94 to -0.4) | -0.28 (-0.47 to -0.09) |
| Estonia | 80.23 (43.92 to 134.25) | 0.01 (0.01 to 0.01) | 7.3 (3.19 to 13.63) | 0.49 (0.45 to 0.53) | -1.96 (-2.78 to -1.14) | 0.28 (0.18 to 0.38) |
| Eswatini | 39.45 (20.18 to 67.25) | 0.13 (0.07 to 0.21) | 9.85 (5.89 to 15.5) | 0.16 (0.12 to 0.21) | 1.15 (0.86 to 1.45) | 0.78 (0.57 to 0.99) |
| Ethiopia | 48.08 (25.04 to 83.17) | 0.03 (0.02 to 0.05) | 5.64 (2.94 to 9.62) | 0.8 (0.72 to 0.88) | -1.55 (-1.72 to -1.38) | -0.07 (-0.13 to 0) |
| Fiji | 68.84 (37.41 to 118.04) | 0.35 (0.23 to 0.53) | 23.89 (15.68 to 34.39) | 0.39 (0.38 to 0.4) | 0.13 (-0.06 to 0.32) | 0.19 (0.03 to 0.34) |
| Finland | 108.49 (87.62 to 131.38) | 0.06 (0.05 to 0.07) | 12 (7.72 to 17.14) | -1.17 (-1.3 to -1.05) | -0.69 (-1.46 to 0.08) | -1.04 (-1.19 to -0.89) |
| France | 81.27 (44.98 to 132.53) | 0.04 (0.03 to 0.04) | 8.58 (4.58 to 14.58) | -0.44 (-0.46 to -0.42) | -1 (-1.35 to -0.65) | -0.58 (-0.66 to -0.5) |
| Gabon | 38.34 (19.3 to 67.89) | 0.06 (0.04 to 0.1) | 6.4 (3.4 to 10.86) | 0.15 (0.13 to 0.16) | -0.33 (-0.79 to 0.13) | -0.09 (-0.25 to 0.07) |
| Gambia | 35.78 (17.66 to 62.55) | 0.02 (0.01 to 0.04) | 4.14 (1.92 to 7.41) | 0.11 (0.1 to 0.12) | 1.05 (0.12 to 2) | 0.24 (-0.14 to 0.61) |
| Georgia | 154.47 (112.3 to 203.9) | 0.14 (0.11 to 0.17) | 19.73 (13.11 to 29.38) | 1.64 (1.47 to 1.81) | 3.04 (2.23 to 3.85) | 2.04 (1.71 to 2.37) |
| Germany | 170.6 (121.68 to 223.95) | 0.05 (0.04 to 0.06) | 16.71 (9.71 to 26.28) | 1.27 (1.09 to 1.46) | -1.42 (-2.3 to -0.54) | 0.66 (0.31 to 1.01) |
| Ghana | 37.28 (18.75 to 63.65) | 0.02 (0.01 to 0.03) | 4.11 (1.78 to 7.98) | 0.27 (0.26 to 0.29) | -0.74 (-0.95 to -0.52) | -0.03 (-0.16 to 0.1) |
| Greece | 74.05 (42.6 to 119.62) | 0.04 (0.03 to 0.05) | 8.29 (4.4 to 14.39) | -0.15 (-0.23 to -0.07) | 0.8 (-0.66 to 2.27) | 0.15 (-0.26 to 0.57) |
| Greenland | 84.17 (45.38 to 139.06) | 0.09 (0.06 to 0.14) | 11.47 (6.81 to 18.57) | -0.58 (-0.65 to -0.51) | -2.15 (-2.37 to -1.94) | -1.29 (-1.43 to -1.14) |
| Grenada | 64.11 (34.8 to 106.02) | 0.08 (0.06 to 0.1) | 9.29 (5.68 to 14.36) | 0.1 (0.08 to 0.12) | -0.62 (-1.14 to -0.1) | -0.19 (-0.47 to 0.08) |
| Guam | 68 (36.68 to 114.82) | 0.36 (0.25 to 0.5) | 24.08 (17.2 to 34.2) | 0.39 (0.35 to 0.43) | 1.29 (1.07 to 1.5) | 1.07 (0.91 to 1.23) |
| Guatemala | 60.86 (33.12 to 101.37) | 0.06 (0.05 to 0.08) | 8.1 (4.69 to 13.11) | 0.17 (0.15 to 0.2) | -0.65 (-1.5 to 0.21) | -0.23 (-0.98 to 0.52) |
| Guinea | 33.38 (16.92 to 58.14) | 0.02 (0.01 to 0.03) | 3.86 (1.75 to 7.27) | 0.13 (0.12 to 0.13) | 0.79 (0.67 to 0.92) | 0.29 (0.22 to 0.36) |
| Guinea-Bissau | 34.31 (17.13 to 59.72) | 0.03 (0.02 to 0.05) | 4.48 (2.37 to 7.89) | 0.1 (0.08 to 0.11) | 0.38 (0.28 to 0.49) | 0.21 (0.15 to 0.27) |
| Guyana | 64.12 (34.71 to 108.64) | 0.12 (0.08 to 0.16) | 11.14 (7.42 to 16.87) | 0.01 (0 to 0.02) | 1.12 (0.45 to 1.79) | 0.53 (0.12 to 0.93) |
| Haiti | 62.51 (33.62 to 105.55) | 0.1 (0.05 to 0.16) | 9.92 (5.69 to 16.24) | 0.06 (0.03 to 0.08) | -0.14 (-0.42 to 0.13) | -0.11 (-0.32 to 0.11) |
| Honduras | 63.18 (32.89 to 105.74) | 0.07 (0.04 to 0.11) | 8.49 (4.52 to 13.74) | 0.02 (0.01 to 0.04) | -0.35 (-0.82 to 0.11) | -0.16 (-0.29 to -0.02) |
| Hungary | 53.16 (28.66 to 88.12) | 0.02 (0.02 to 0.02) | 5.5 (2.59 to 10.31) | -0.44 (-0.46 to -0.41) | -2.15 (-3.32 to -0.97) | -0.8 (-1.21 to -0.38) |
| Iceland | 103.42 (61.69 to 149.03) | 0.06 (0.05 to 0.08) | 12.01 (7.1 to 18.76) | 0.85 (0.77 to 0.92) | 1.15 (0.82 to 1.48) | 0.96 (0.79 to 1.14) |
| India | 31.87 (15.64 to 57.56) | 0.04 (0.03 to 0.05) | 4.63 (2.94 to 7.2) | 0.1 (0.08 to 0.11) | 0.65 (0.59 to 0.71) | 0.31 (0.29 to 0.33) |
| Indonesia | 64.09 (34.26 to 110.24) | 0.1 (0.08 to 0.14) | 10.69 (7.37 to 15.76) | 0.14 (0.11 to 0.17) | 0.07 (-0.04 to 0.18) | 0.11 (0.05 to 0.16) |
| Iran (Islamic Republic of) | 32.14 (15.56 to 56.95) | 0.02 (0.02 to 0.03) | 3.63 (2.09 to 6.22) | 0.27 (0.24 to 0.31) | -0.73 (-0.91 to -0.55) | -0.04 (-0.12 to 0.05) |
| Iraq | 36.84 (17.66 to 65.67) | 0.11 (0.07 to 0.18) | 8.77 (5.35 to 13.02) | 0.29 (0.27 to 0.31) | -0.55 (-0.76 to -0.35) | -0.28 (-0.45 to -0.11) |
| Ireland | 74.72 (42.73 to 120.12) | 0.04 (0.03 to 0.04) | 8.09 (4.36 to 13.71) | -0.53 (-0.54 to -0.51) | -0.84 (-1.31 to -0.37) | -0.61 (-0.83 to -0.38) |
| Israel | 106.71 (59.34 to 172.77) | 0.03 (0.03 to 0.04) | 10.56 (5.32 to 18.34) | 0.37 (0.32 to 0.42) | -0.9 (-1.41 to -0.4) | 0.22 (0.04 to 0.4) |
| Italy | 66.15 (35.82 to 110.86) | 0.04 (0.04 to 0.04) | 7.46 (4.56 to 11.88) | -0.46 (-0.54 to -0.38) | -0.93 (-1.36 to -0.49) | -0.58 (-0.72 to -0.43) |
| Jamaica | 64.48 (34.44 to 108.97) | 0.08 (0.05 to 0.11) | 9.27 (5.45 to 14.72) | 0.14 (0.12 to 0.16) | 1.01 (-0.9 to 2.96) | 0.46 (-0.05 to 0.96) |
| Japan | 56.04 (29.9 to 94.88) | 0.04 (0.03 to 0.04) | 6.49 (4.01 to 10.51) | -1.19 (-1.29 to -1.09) | -0.64 (-1.38 to 0.11) | -0.99 (-1.33 to -0.65) |
| Jordan | 35.85 (17.44 to 62.81) | 0.03 (0.02 to 0.05) | 4.66 (2.38 to 8.01) | 0.42 (0.4 to 0.45) | -1.86 (-2.32 to -1.39) | -0.64 (-0.85 to -0.42) |
| Kazakhstan | 49.51 (25.62 to 83.71) | 0.03 (0.02 to 0.04) | 5.73 (2.93 to 10.47) | 0.12 (0.06 to 0.18) | -0.56 (-1.78 to 0.67) | -0.03 (-0.33 to 0.27) |
| Kenya | 42.34 (21.57 to 73.94) | 0.04 (0.03 to 0.06) | 5.5 (3.31 to 8.96) | 0.06 (0.05 to 0.07) | 0.75 (0.55 to 0.95) | 0.3 (0.23 to 0.36) |
| Kiribati | 61.36 (32.86 to 103.96) | 0.29 (0.18 to 0.44) | 20.21 (12.99 to 29.46) | 0.12 (0.11 to 0.13) | 0.33 (0.24 to 0.42) | 0.3 (0.27 to 0.32) |
| Kuwait | 35.86 (17.47 to 62.25) | 0.04 (0.03 to 0.04) | 4.74 (2.53 to 7.93) | 0.04 (-0.04 to 0.11) | -0.53 (-1.41 to 0.35) | -0.2 (-0.55 to 0.15) |
| Kyrgyzstan | 42.57 (22.02 to 72.14) | 0.03 (0.03 to 0.04) | 5.4 (2.87 to 9.48) | 0.06 (-0.01 to 0.13) | 0.26 (0 to 0.53) | 0.13 (0.04 to 0.22) |
| Lao People's Democratic Republic | 61.4 (33.66 to 102.1) | 0.11 (0.07 to 0.16) | 10.7 (6.52 to 17.31) | 0.12 (0.1 to 0.14) | -0.33 (-0.4 to -0.26) | -0.1 (-0.19 to -0.01) |
| Latvia | 96.57 (60.59 to 136.5) | 0.02 (0.02 to 0.02) | 9.09 (4.62 to 15.1) | 1.23 (1.12 to 1.35) | 0.78 (-0.96 to 2.56) | 1.2 (1 to 1.41) |
| Lebanon | 33.79 (16.65 to 59.3) | 0.04 (0.03 to 0.05) | 4.59 (2.48 to 7.77) | 0.38 (0.33 to 0.43) | -3.44 (-3.79 to -3.08) | -1.76 (-2.05 to -1.48) |
| Lesotho | 36.34 (18.52 to 62.78) | 0.11 (0.06 to 0.17) | 8.35 (4.89 to 13.31) | 0.16 (0.14 to 0.18) | 2.67 (2.28 to 3.06) | 1.5 (1.25 to 1.75) |
| Liberia | 35.2 (17.89 to 62.33) | 0.02 (0.01 to 0.03) | 3.99 (1.7 to 7.44) | 0.08 (0.06 to 0.1) | 0.64 (0.37 to 0.9) | 0.17 (0.01 to 0.34) |
| Libya | 35.66 (17.47 to 62.56) | 0.04 (0.02 to 0.08) | 5.03 (2.24 to 8.93) | 0.17 (0.14 to 0.21) | 0.98 (0.54 to 1.43) | 0.54 (0.49 to 0.59) |
| Lithuania | 80.73 (44.47 to 135.66) | 0.02 (0.02 to 0.02) | 7.76 (3.73 to 14.43) | 0.46 (0.44 to 0.48) | -0.17 (-0.94 to 0.6) | 0.41 (0.15 to 0.68) |
| Luxembourg | 78.87 (51.89 to 109.81) | 0.03 (0.02 to 0.03) | 7.89 (4.52 to 12.92) | -0.11 (-0.21 to -0.01) | -2.76 (-4.16 to -1.34) | -0.77 (-1.01 to -0.53) |
| Madagascar | 42.45 (21.59 to 75.03) | 0.11 (0.07 to 0.17) | 9.21 (5.48 to 14.34) | 0.14 (0.12 to 0.16) | -0.16 (-0.41 to 0.09) | -0.05 (-0.11 to 0.02) |
| Malawi | 45.16 (22.91 to 78.49) | 0.05 (0.03 to 0.09) | 6.34 (3.3 to 10.51) | 0.24 (0.22 to 0.26) | 1.08 (0.82 to 1.33) | 0.59 (0.52 to 0.65) |
| Malaysia | 67.73 (36.6 to 116.61) | 0.08 (0.06 to 0.1) | 9.7 (5.83 to 15.22) | 0.24 (0.17 to 0.31) | 0.54 (0.32 to 0.77) | 0.33 (0.14 to 0.52) |
| Maldives | 63.61 (34.7 to 107.72) | 0.04 (0.02 to 0.07) | 7.41 (3.54 to 13.28) | 0.19 (0.16 to 0.23) | -1.97 (-2.56 to -1.37) | -0.47 (-0.68 to -0.25) |
| Mali | 33.39 (16.86 to 57.89) | 0.02 (0.01 to 0.03) | 3.71 (1.63 to 6.91) | 0.12 (0.1 to 0.14) | 0.19 (-0.04 to 0.43) | 0.16 (0.09 to 0.22) |
| Malta | 76.32 (51.07 to 104.5) | 0.04 (0.04 to 0.05) | 8.7 (5.22 to 13.85) | -0.01 (-0.12 to 0.11) | 1.05 (0.27 to 1.84) | 0.25 (-0.12 to 0.62) |
| Marshall Islands | 59.24 (30.78 to 100.37) | 0.68 (0.38 to 1.06) | 39.61 (23.87 to 59.96) | 0.19 (0.17 to 0.21) | 0.93 (0.86 to 1.01) | 0.83 (0.76 to 0.89) |
| Mauritania | 35.7 (18.06 to 63.32) | 0.02 (0.01 to 0.03) | 3.95 (1.61 to 7.61) | 0.19 (0.18 to 0.2) | -0.28 (-0.43 to -0.13) | 0.13 (-0.03 to 0.3) |
| Mauritius | 64.47 (34.82 to 107.84) | 0.12 (0.1 to 0.14) | 11.47 (7.67 to 17.36) | 0.1 (0.07 to 0.13) | 0.65 (-0.66 to 1.98) | 0.38 (-0.35 to 1.11) |
| Mexico | 62.22 (32.88 to 106.17) | 0.07 (0.06 to 0.08) | 8.7 (5.76 to 13.19) | 0.16 (0.13 to 0.18) | 0.11 (-0.14 to 0.36) | 0.12 (0.02 to 0.23) |
| Micronesia (Federated States of) | 59.76 (31.83 to 101.01) | 0.66 (0.4 to 0.98) | 38.94 (24.26 to 56.24) | 0.04 (0.03 to 0.06) | 0.28 (0.27 to 0.3) | 0.26 (0.21 to 0.3) |
| Monaco | 77.82 (43.06 to 128.06) | 0.04 (0.02 to 0.07) | 8.84 (4.82 to 15.35) | -0.44 (-0.55 to -0.32) | -0.13 (-0.28 to 0.01) | -0.39 (-0.51 to -0.26) |
| Mongolia | 46.37 (23.48 to 79.13) | 0.03 (0.02 to 0.04) | 5.3 (2.57 to 9.48) | 0.24 (0.2 to 0.27) | 0.24 (-0.51 to 1.01) | 0.22 (-0.12 to 0.56) |
| Montenegro | 54.08 (29.02 to 91.3) | 0.09 (0.06 to 0.14) | 9.27 (5.58 to 14.1) | -0.09 (-0.14 to -0.04) | -0.91 (-1.84 to 0.03) | -0.48 (-1.06 to 0.11) |
| Morocco | 34.05 (16.6 to 59.87) | 0.04 (0.02 to 0.07) | 4.58 (2.35 to 8.25) | 0.21 (0.17 to 0.26) | 0.17 (0.01 to 0.33) | 0.15 (0.08 to 0.22) |
| Mozambique | 43.49 (22.14 to 74.37) | 0.08 (0.04 to 0.14) | 7.37 (3.74 to 12.49) | 0.2 (0.19 to 0.2) | 1.71 (1.59 to 1.83) | 0.93 (0.81 to 1.04) |
| Myanmar | 61.86 (33.23 to 103.09) | 0.11 (0.08 to 0.17) | 11.06 (6.75 to 17.4) | 0.01 (-0.04 to 0.06) | -0.53 (-0.59 to -0.48) | -0.28 (-0.36 to -0.2) |
| Namibia | 37.15 (18.73 to 64.94) | 0.09 (0.05 to 0.14) | 7.37 (4.24 to 12.16) | 0.05 (0.05 to 0.06) | 0.57 (0.27 to 0.87) | 0.41 (0.27 to 0.55) |
| Nauru | 68.48 (36.6 to 114.6) | 0.77 (0.41 to 1.18) | 45.65 (25.06 to 67.42) | 0.17 (0.15 to 0.19) | 0.21 (0.12 to 0.3) | 0.22 (0.11 to 0.33) |
| Nepal | 30.77 (14.95 to 53.38) | 0.03 (0.02 to 0.05) | 4.27 (2.16 to 7.44) | -0.04 (-0.07 to -0.02) | 0.94 (0.76 to 1.12) | 0.32 (0.23 to 0.4) |
| Netherlands | 82.15 (45.57 to 132.68) | 0.03 (0.03 to 0.04) | 8.63 (4.47 to 15.05) | -0.32 (-0.36 to -0.29) | -0.82 (-1.2 to -0.44) | -0.46 (-0.68 to -0.25) |
| New Zealand | 100.56 (61.69 to 151.69) | 0.05 (0.05 to 0.06) | 10.92 (6.56 to 17.67) | 0.4 (0.33 to 0.46) | -0.04 (-0.42 to 0.34) | 0.27 (0.06 to 0.48) |
| Nicaragua | 63.48 (34.25 to 105.78) | 0.05 (0.03 to 0.07) | 7.6 (3.97 to 12.42) | 0.05 (0.04 to 0.06) | -0.66 (-1.04 to -0.29) | -0.23 (-0.42 to -0.05) |
| Niger | 33.26 (16.53 to 56.55) | 0.01 (0 to 0.02) | 3.36 (1.27 to 6.56) | 0.1 (0.08 to 0.12) | 0.06 (-0.09 to 0.2) | 0.1 (-0.03 to 0.23) |
| Nigeria | 35.47 (17.66 to 61.71) | 0.02 (0.01 to 0.02) | 3.75 (2.03 to 6.58) | 0.35 (0.33 to 0.36) | -0.57 (-0.72 to -0.42) | 0.1 (0.01 to 0.2) |
| Niue | 68.02 (37.35 to 113.24) | 0.47 (0.31 to 0.69) | 29.85 (20.3 to 42.43) | 0.28 (0.26 to 0.3) | 0.18 (-0.07 to 0.44) | 0.23 (0 to 0.46) |
| North Macedonia | 53.09 (28.92 to 91.05) | 0.02 (0.01 to 0.04) | 5.54 (2.46 to 9.94) | 0.03 (-0.06 to 0.12) | -0.37 (-0.76 to 0.03) | -0.08 (-0.32 to 0.17) |
| Northern Mariana Islands | 67.51 (36.19 to 114.58) | 0.42 (0.28 to 0.63) | 27 (18.8 to 39.01) | 0.19 (0.1 to 0.28) | -0.25 (-1.69 to 1.22) | -0.13 (-1.15 to 0.9) |
| Norway | 73.08 (40.43 to 121.84) | 0.02 (0.01 to 0.02) | 6.96 (3.75 to 12.01) | -0.27 (-0.32 to -0.23) | -1.91 (-3.36 to -0.44) | -0.5 (-0.73 to -0.26) |
| Oman | 36.06 (17.42 to 62.11) | 0.05 (0.03 to 0.07) | 5.5 (3.02 to 9.39) | 0.58 (0.55 to 0.62) | -0.97 (-1.42 to -0.52) | -0.25 (-0.45 to -0.05) |
| Pakistan | 33 (16.44 to 58.48) | 0.05 (0.03 to 0.07) | 5.18 (3.18 to 8) | 0.08 (0.07 to 0.09) | 1.51 (1.41 to 1.6) | 0.66 (0.61 to 0.71) |
| Palau | 65.93 (35.87 to 111.53) | 0.3 (0.21 to 0.43) | 21.15 (14.59 to 29.36) | 0.32 (0.3 to 0.35) | -0.23 (-0.44 to -0.02) | -0.1 (-0.27 to 0.06) |
| Palestine | 31.75 (15.63 to 55.86) | 0.04 (0.03 to 0.06) | 4.66 (2.6 to 7.66) | 0.26 (0.22 to 0.3) | -0.97 (-1.32 to -0.62) | -0.37 (-0.42 to -0.33) |
| Panama | 66.09 (35.91 to 110.44) | 0.06 (0.04 to 0.07) | 8.31 (4.74 to 13.53) | 0.13 (0.1 to 0.15) | 0.7 (0.11 to 1.29) | 0.31 (0.12 to 0.5) |
| Papua New Guinea | 55.25 (29.3 to 92.71) | 0.34 (0.19 to 0.54) | 22.29 (13.37 to 33.89) | 0.06 (0.03 to 0.08) | 0.08 (-0.14 to 0.3) | 0.07 (-0.11 to 0.25) |
| Paraguay | 113.14 (66.38 to 182.94) | 0.07 (0.05 to 0.1) | 12.83 (7.32 to 20.92) | -0.08 (-0.1 to -0.06) | 0.25 (0.09 to 0.41) | -0.02 (-0.11 to 0.07) |
| Peru | 63.91 (35.13 to 108.47) | 0.07 (0.04 to 0.09) | 8.55 (4.78 to 13.93) | 0.39 (0.36 to 0.43) | -1.03 (-2.06 to 0.01) | -0.26 (-0.76 to 0.23) |
| Philippines | 57.55 (30.68 to 98.35) | 0.13 (0.1 to 0.16) | 11.65 (8.37 to 15.81) | 0.05 (0.03 to 0.08) | 0.56 (0.27 to 0.86) | 0.33 (0.17 to 0.49) |
| Poland | 73.32 (40.4 to 122.06) | 0.02 (0.02 to 0.02) | 7.22 (4.07 to 12.24) | 1.2 (1.11 to 1.3) | -2.52 (-3.03 to -2.01) | 0.36 (0.08 to 0.64) |
| Portugal | 58.21 (33.55 to 91.79) | 0.03 (0.03 to 0.04) | 6.42 (3.42 to 11.02) | -1.07 (-1.24 to -0.9) | -1.26 (-2.18 to -0.32) | -1.1 (-1.27 to -0.92) |
| Puerto Rico | 68.16 (36.73 to 115.09) | 0.07 (0.05 to 0.08) | 8.95 (5.17 to 14.62) | 0.08 (0.05 to 0.11) | -0.64 (-1.53 to 0.25) | -0.21 (-0.63 to 0.22) |
| Qatar | 40.21 (19.58 to 69.94) | 0.01 (0.01 to 0.03) | 3.86 (1.24 to 8.1) | 0.29 (0.26 to 0.32) | -2.02 (-3.13 to -0.9) | -0.37 -0.84 to 0.09) |
| Republic of Korea | 224.38 (158.2 to 297.45) | 0.04 (0.03 to 0.05) | 20.8 (12.13 to 32.35) | 1.67 (1.17 to 2.17) | -2.48 (-2.72 to -2.24) | 0.99 (0.63 to 1.36) |
| Republic of Moldova | 79.4 (43.04 to 134.12) | 0.02 (0.02 to 0.02) | 7.54 (3.45 to 13.82) | 0.49 (0.47 to 0.52) | 0.05 (-0.65 to 0.76) | 0.44 (0.25 to 0.63) |
| Romania | 40.92 (26.45 to 59.41) | 0.02 (0.02 to 0.02) | 4.47 (2.32 to 8.16) | -0.79 (-0.84 to -0.73) | -0.57 (-0.89 to -0.24) | -0.7 (-0.89 to -0.5) |
| Russian Federation | 70.96 (39.15 to 118.14) | 0.03 (0.03 to 0.03) | 7.29 (4.32 to 11.99) | 0.29 (0.25 to 0.32) | 1.38 (0.73 to 2.04) | 0.45 (0.29 to 0.6) |
| Rwanda | 43.67 (21.76 to 76.25) | 0.04 (0.02 to 0.08) | 5.73 (2.56 to 10.32) | 0.07 (0.07 to 0.08) | -2.36 (-2.75 to -1.96) | -1.03 (-1.25 to -0.8) |
| Saint Kitts and Nevis | 64.03 (34.73 to 107.38) | 0.05 (0.04 to 0.07) | 7.87 (4.2 to 13.13) | 0.03 (-0.04 to 0.1) | -2.81 (-3.39 to -2.22) | -1.25 (-1.52 to -0.98) |
| Saint Lucia | 65.42 (35.38 to 109.32) | 0.07 (0.06 to 0.09) | 8.97 (5.5 to 13.74) | -0.02 (-0.05 to 0) | -1.61 (-2.73 to -0.48) | -0.79 (-1.22 to -0.36) |
| Saint Vincent and the Grenadines | 63.8 (34.92 to 106.9) | 0.1 (0.08 to 0.12) | 10.13 (6.7 to 15.47) | 0.13 (0.1 to 0.16) | -2.14 (-3.35 to -0.91) | -1.12 (-1.69 to -0.55) |
| Samoa | 64.19 (35.27 to 108.69) | 0.48 (0.3 to 0.74) | 29.93 (19.56 to 44.68) | 0.1 (0.07 to 0.13) | 0.64 (0.61 to 0.66) | 0.54 (0.5 to 0.59) |
| San Marino | 77.53 (41.75 to 128.18) | 0.02 (0.01 to 0.03) | 7.46 (3.4 to 13.41) | -0.56 (-0.6 to -0.52) | -2.2 (-2.48 to -1.91) | -0.82 (-0.94 to -0.7) |
| Sao Tome and Principe | 37.26 (18.87 to 65.78) | 0.02 (0.01 to 0.03) | 4.19 (1.76 to 7.83) | 0.26 (0.25 to 0.27) | 0.48 (0.11 to 0.86) | 0.31 (0.1 to 0.53) |
| Saudi Arabia | 36.45 (17.85 to 62.32) | 0.08 (0.05 to 0.12) | 7.15 (4.23 to 11.66) | 0.42 (0.37 to 0.47) | 0.99 (0.82 to 1.17) | 0.7 (0.58 to 0.82) |
| Senegal | 36 (18.05 to 62.7) | 0.02 (0.01 to 0.03) | 4.01 (1.68 to 7.19) | 0.11 (0.08 to 0.13) | 0.56 (0.41 to 0.71) | 0.24 (0.18 to 0.3) |
| Serbia | 42.26 (24.76 to 64.28) | 0.02 (0.01 to 0.03) | 4.51 (2.07 to 7.99) | -0.56 (-0.62 to -0.49) | -1.05 (-1.3 to -0.79) | -0.65 (-0.91 to -0.4) |
| Seychelles | 65.54 (35.09 to 109.92) | 0.11 (0.08 to 0.15) | 11.31 (7.18 to 18.07) | 0.09 (0.05 to 0.14) | -0.5 (-1.37 to 0.37) | -0.23 (-0.58 to 0.12) |
| Sierra Leone | 37.3 (18.68 to 65.67) | 0.02 (0.01 to 0.03) | 4.07 (1.73 to 7.51) | -0.06 (-0.07 to -0.06) | 0.85 (0.55 to 1.16) | 0.15 (0.06 to 0.23) |
| Singapore | 90.36 (49.84 to 141.12) | 0.01 (0.01 to 0.01) | 8.28 (3.68 to 15.34) | -0.47 (-0.55 to -0.4) | -3.44 (-3.98 to -2.9) | -0.8 (-0.95 to -0.64) |
| Slovakia | 70.7 (41.65 to 111.13) | 0.04 (0.02 to 0.05) | 7.75 (4.06 to 13.06) | -0.14 (-0.22 to -0.07) | -0.98 (-1.54 to -0.41) | -0.41 (-0.55 to -0.26) |
| Slovenia | 62.12 (41.09 to 86.7) | 0.01 (0.01 to 0.01) | 5.7 (2.7 to 10.11) | 0.29 (0.22 to 0.35) | -3.02 (-3.35 to -2.68) | -0.2 (-0.4 to -0.01) |
| Solomon Islands | 58.05 (30.76 to 98.19) | 0.32 (0.18 to 0.53) | 21.31 (12.96 to 32.56) | 0.13 (0.11 to 0.15) | 1.03 (0.77 to 1.3) | 0.8 (0.59 to 1.01) |
| Somalia | 40.78 (20.74 to 71.12) | 0.04 (0.01 to 0.09) | 5.44 (2.3 to 9.98) | 0.14 (0.12 to 0.15) | -0.89 (-1.08 to -0.7) | -0.29 (-0.47 to -0.12) |
| South Africa | 39.61 (20.22 to 68.65) | 0.13 (0.1 to 0.17) | 9.58 (7.05 to 13.18) | -0.03 (-0.04 to -0.01) | -0.72 (-1.92 to 0.5) | -0.55 (-1.45 to 0.36) |
| South Sudan | 42.79 (21.63 to 74.6) | 0.05 (0.02 to 0.1) | 5.94 (2.74 to 10.49) | 0.19 (0.17 to 0.21) | -0.12 (-0.4 to 0.17) | 0.06 (-0.11 to 0.23) |
| Spain | 106.32 (65.6 to 162.14) | 0.03 (0.02 to 0.03) | 10.37 (5.44 to 16.84) | 0.25 (-0.22 to 0.72) | -1.65 (-1.9 to -1.4) | -0.07 (-0.47 to 0.34) |
| Sri Lanka | 62.77 (34.01 to 104.9) | 0.05 (0.03 to 0.08) | 7.98 (4.18 to 13.23) | 0.15 (0.1 to 0.2) | -0.44 (-1.21 to 0.34) | 0 (-0.18 to 0.18) |
| Sudan | 33.91 (16.52 to 59.48) | 0.04 (0.02 to 0.07) | 4.83 (2.32 to 8.27) | 0.39 (0.37 to 0.42) | -0.09 (-0.15 to -0.03) | 0.17 (0.11 to 0.22) |
| Suriname | 62.81 (34.24 to 104.92) | 0.1 (0.07 to 0.15) | 10.41 (6.46 to 16.07) | 0.01 (-0.01 to 0.04) | -0.07 (-0.85 to 0.71) | -0.06 (-0.44 to 0.32) |
| Sweden | 144.28 (82.18 to 233.51) | 0.02 (0.02 to 0.03) | 13.25 (6.63 to 23.33) | 0.82 (0.58 to 1.07) | 0.26 (-0.74 to 1.27) | 0.68 (0.49 to 0.87) |
| Switzerland | 51.74 (36.06 to 71.75) | 0.01 (0.01 to 0.02) | 5.04 (2.47 to 8.71) | -0.23 (-0.39 to -0.08) | -2.01 (-2.56 to -1.46) | -0.47 (-0.79 to -0.14) |
| Syrian Arab Republic | 32.28 (15.43 to 56.53) | 0.04 (0.02 to 0.07) | 4.56 (2.17 to 8.08) | 0.13 (0.09 to 0.16) | -0.35 (-0.86 to 0.16) | -0.09 (-0.36 to 0.19) |
| Taiwan (Province of China) | 78.92 (43.88 to 128.36) | 0.02 (0.02 to 0.02) | 7.67 (3.55 to 14.06) | -0.18 (-0.24 to -0.12) | -0.09 (-0.5 to 0.31) | -0.16 (-0.35 to 0.02) |
| Tajikistan | 41.84 (21.5 to 72.24) | 0.01 (0.01 to 0.02) | 4.21 (1.82 to 8.17) | 0.08 (0.06 to 0.1) | -0.7 (-0.83 to -0.57) | -0.04 (-0.17 to 0.09) |
| Thailand | 60.29 (32.43 to 102.04) | 0.12 (0.08 to 0.17) | 11.36 (6.85 to 17.32) | 0.15 (0.11 to 0.19) | 0.38 (-0.03 to 0.79) | 0.31 (0.06 to 0.57) |
| Timor-Leste | 61.34 (32.9 to 102.1) | 0.07 (0.05 to 0.11) | 8.9 (4.97 to 15.12) | 0 (0.05 to 0.15) | 0.26 (-0.16 to 0.67) | 0.14 (-0.02 to 0.3) |
| Togo | 35.94 (18.02 to 62.96) | 0.02 (0.01 to 0.03) | 4.12 (1.79 to 7.58) | 0.11 (0.07 to 0.16) | 0.63 (0.42 to 0.84) | 0.22 (0.03 to 0.42) |
| Tokelau | 63.38 (34.18 to 105.41) | 0.52 (0.32 to 0.82) | 32.38 (21.02 to 48.11) | 0.38 (0.33 to 0.42) | 0.21 (-0.02 to 0.44) | 0.27 (0.08 to 0.45) |
| Tonga | 65.98 (35.33 to 112.59) | 0.28 (0.16 to 0.46) | 19.8 (12.31 to 30.44) | 0.32 (0.3 to 0.34) | 0.27 (0.05 to 0.5) | 0.31 (0.13 to 0.5) |
| Trinidad and Tobago | 70.34 (38.58 to 117.23) | 0.1 (0.07 to 0.14) | 11.07 (6.79 to 16.96) | 0.13 (0.1 to 0.15) | 0.31 (-0.63 to 1.27) | 0.2 (-0.27 to 0.66) |
| Tunisia | 32.37 (15.76 to 56.54) | 0.03 (0.02 to 0.06) | 4.2 (2.05 to 7.42) | 0.27 (0.25 to 0.28) | 0.01 (-0.11 to 0.13) | 0.17 (-0.02 to 0.36) |
| Turkey | 33.89 (23.84 to 45.38) | 0.03 (0.02 to 0.05) | 4.51 (2.62 to 7.06) | 0.16 (0.1 to 0.22) | -1.66 (-2.01 to -1.31) | -0.68 (-0.91 to -0.45) |
| Turkmenistan | 49.51 (25.89 to 84.18) | 0.05 (0.03 to 0.06) | 6.49 (3.5 to 10.98) | 0.49 (0.45 to 0.53) | 1.19 (0.62 to 1.75) | 0.74 (0.53 to 0.95) |
| Tuvalu | 62.29 (34.05 to 104.25) | 0.54 (0.36 to 0.76) | 32.8 (21.83 to 46.56) | 0.37 (0.35 to 0.39) | 0 (-0.05 to 0.05) | 0.07 (-0.01 to 0.15) |
| Uganda | 44.65 (22.46 to 76.92) | 0.04 (0.02 to 0.08) | 5.89 (2.85 to 10.28) | 0.04 (0 to 0.08) | 0.15 (-0.04 to 0.34) | 0.06 (-0.1 to 0.22) |
| Ukraine | 63.83 (34.54 to 106.44) | 0.02 (0.01 to 0.03) | 6.16 (3.11 to 11.08) | 0.18 (0.17 to 0.19) | 1.63 (0.53 to 2.73) | 0.34 (0.1 to 0.59) |
| United Arab Emirates | 40.24 (19.95 to 68.64) | 0.02 (0.01 to 0.03) | 4.37 (1.79 to 8.15) | 0.09 (0.04 to 0.14) | -2.13 (-2.57 to -1.69) | -0.74 (-1.21 to -0.27) |
| United Kingdom | 79.83 (48.38 to 119.57) | 0.03 (0.02 to 0.03) | 7.99 (4.89 to 12.67) | 0.62 (0.56 to 0.68) | 0.54 (-0.12 to 1.2) | 0.59 (0.48 to 0.7) |
| United Republic of Tanzania | 44.87 (23.08 to 77.96) | 0.04 (0.02 to 0.08) | 5.83 (2.83 to 10.19) | 0.38 (0.36 to 0.4) | -0.5 (-0.72 to -0.28) | 0.02 (-0.09 to 0.13) |
| United States of America | 93.13 (74.55 to 117.38) | 0.07 (0.07 to 0.07) | 11.17 (8.59 to 14.56) | 0.05 (-0.04 to 0.14) | 1.9 (1.62 to 2.19) | 0.52 (0.4 to 0.63) |
| United States Virgin Islands | 67.72 (36.6 to 112.84) | 0.08 (0.05 to 0.12) | 9.63 (5.53 to 15.26) | 0.09 (0.07 to 0.11) | -1.02 (-1.6 to -0.43) | -0.42 (-0.7 to -0.13) |
| Uruguay | 47.79 (25.17 to 82.05) | 0.05 (0.04 to 0.05) | 6.35 (3.67 to 10.43) | 0.05 (0.02 to 0.09) | 0.46 (-0.14 to 1.06) | 0.2 (-0.07 to 0.48) |
| Uzbekistan | 42.59 (22.09 to 73.18) | 0.02 (0.01 to 0.02) | 4.47 (1.95 to 8.36) | 0.2 (0.13 to 0.28) | 0.53 (0.23 to 0.84) | 0.32 (0.17 to 0.46) |
| Vanuatu | 66.7 (35.77 to 112.9) | 0.44 (0.27 to 0.67) | 28.3 (18.58 to 40.69) | 0.09 (0.06 to 0.11) | 0.7 (0.43 to 0.97) | 0.55 (0.34 to 0.76) |
| Venezuela (Bolivarian Republic of) | 67.3 (36.11 to 111.76) | 0.07 (0.05 to 0.09) | 8.94 (5.19 to 14.28) | -0.13 (-0.14 to -0.12) | 0.62 (-0.03 to 1.28) | 0.08 (-0.18 to 0.35) |
| Viet Nam | 65.13 (35.35 to 110.16) | 0.08 (0.05 to 0.13) | 9.6 (5.19 to 15.62) | 0.35 (0.3 to 0.4) | 0.12 (0.06 to 0.19) | 0.25 (0.11 to 0.39) |
| Yemen | 30.7 (15.21 to 52.41) | 0.04 (0.02 to 0.06) | 4.47 (2.2 to 7.85) | 0.22 (0.17 to 0.26) | 0.26 (-0.17 to 0.69) | 0.23 (0.06 to 0.39) |
| Zambia | 41.57 (21.14 to 72.59) | 0.1 (0.04 to 0.22) | 8.37 (3.74 to 15.34) | 0.13 (0.12 to 0.14) | 1.15 (1 to 1.31) | 0.74 (0.58 to 0.9) |
| Zimbabwe | 37.5 (18.9 to 64.58) | 0.11 (0.06 to 0.17) | 8.69 (5.11 to 13.36) | 0.11 (0.09 to 0.13) | 2.71 (1.98 to 3.45) | 1.58 (1.15 to 2.01) |

AAPC=average annual percentage change; DALYs=Disability-Adjusted Life Years; ASMR=Age-standardized mortality rates; ASDR=Age-standardized DALYs.


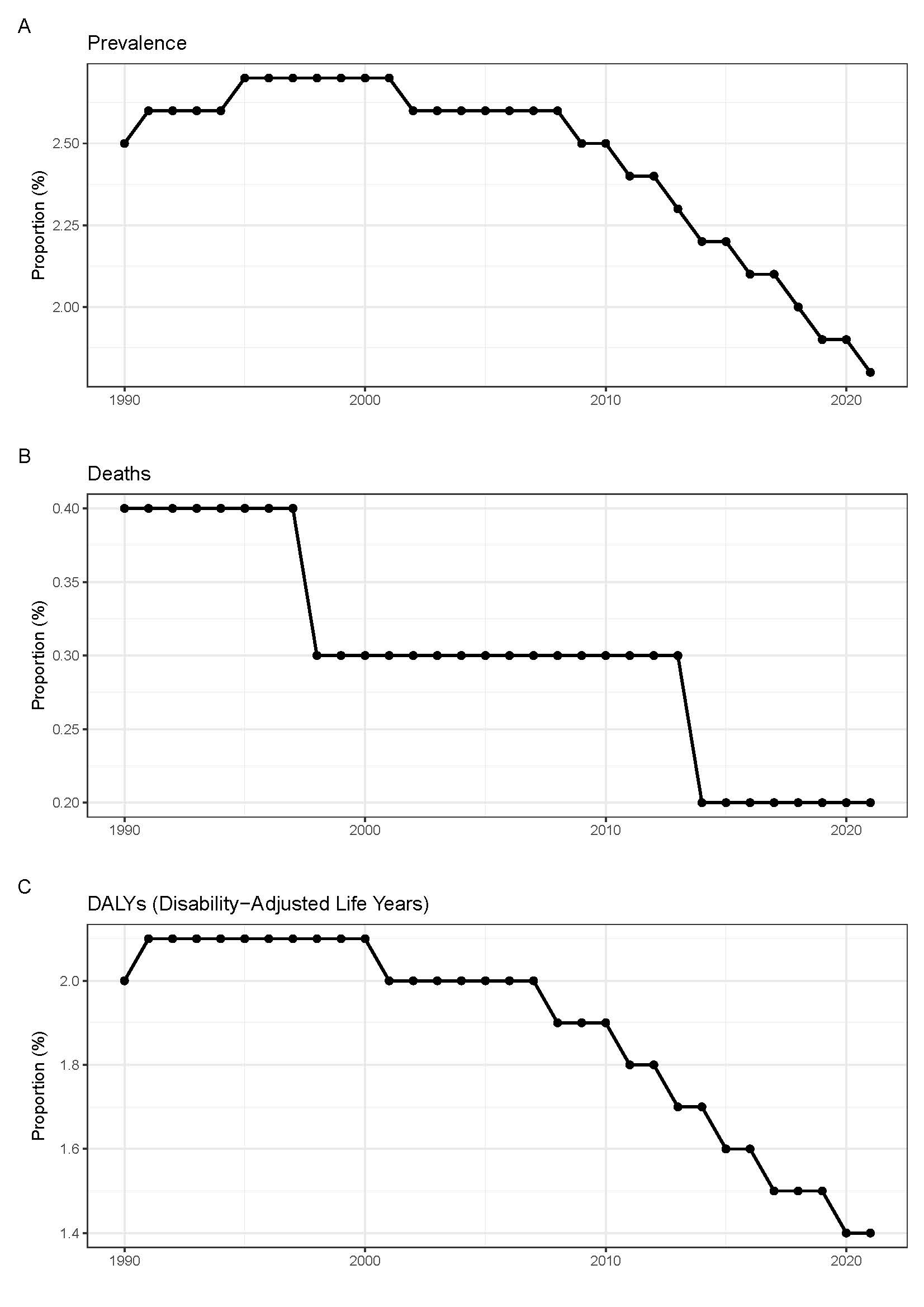


**Figure S1.** (A) Change in the proportion of prevalence among atrial fibrillation patients aged 30-45 years relative to all atrial fibrillation patients, 1990 to 2021. (B) Change in the proportion of deaths among atrial fibrillation patients aged 30-45 years relative to all atrial fibrillation deaths, 1990 to 2021. (C) Change in the proportion of Disability-Adjusted Life Years (DALYs) among atrial fibrillation patients aged 30 - 45 years relative to all atrial fibrillation DALYs, 1990 to 2021.


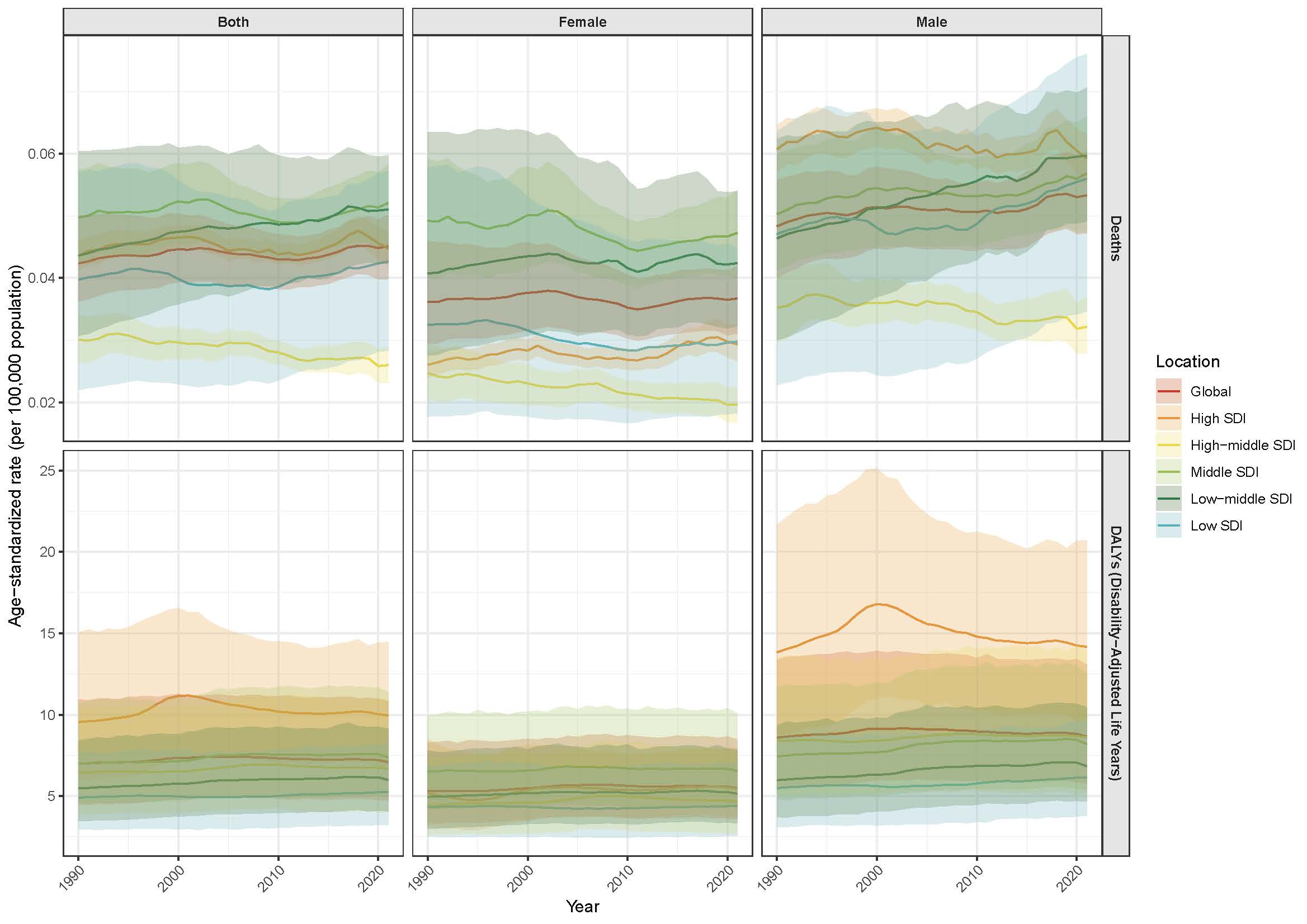


**Figure S2.** Temporal trend of ASMR and ASDR of atrial fibrillation in patients aged 30-45 years from 1990 to 2021 at global and socio-demographic index (SDI) levels by sex.


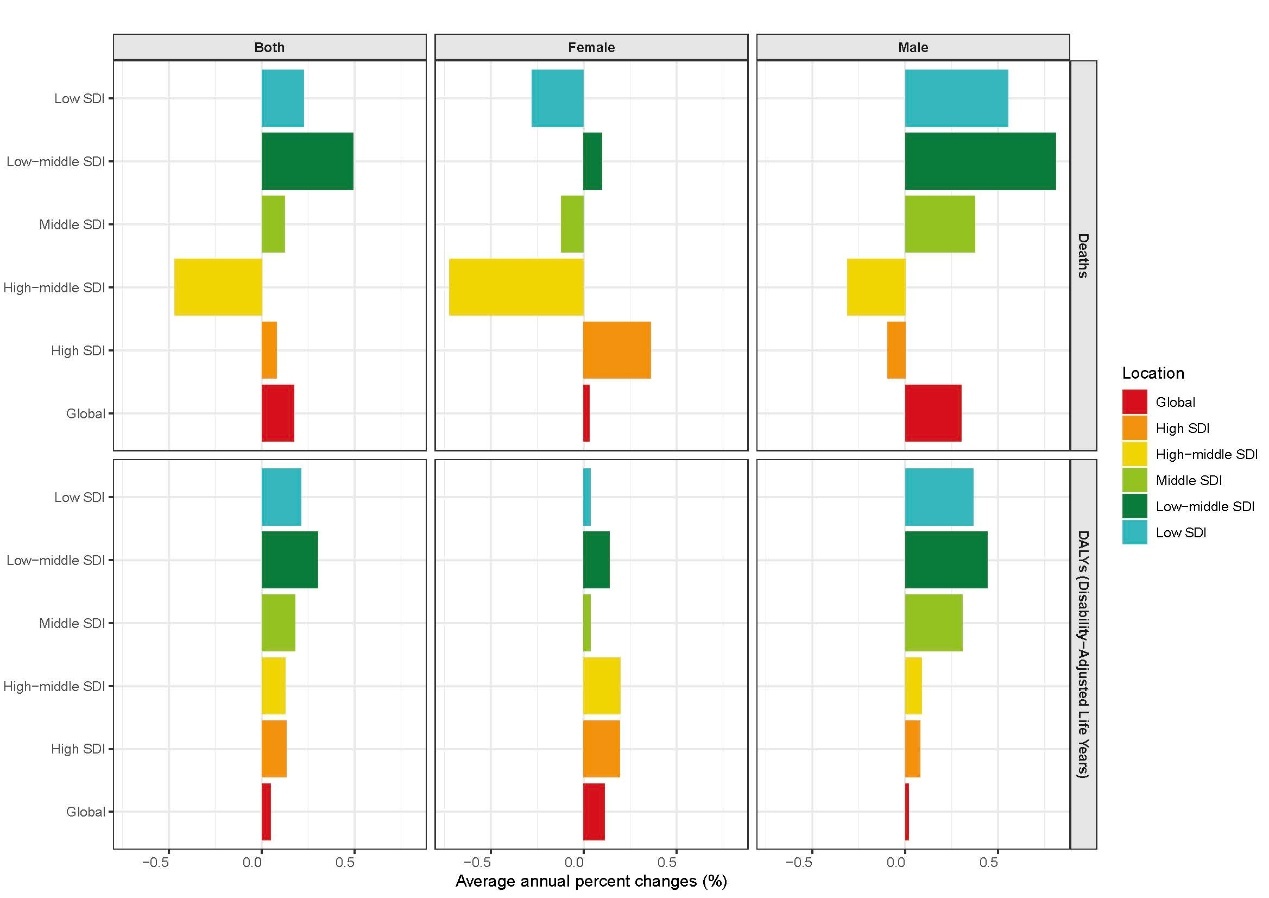


**Figure S3.** AAPC of ASMR and ASDR of atrial fibrillation in patients aged 30-45 years from 1990 to 2021 at SDI levels by sex.


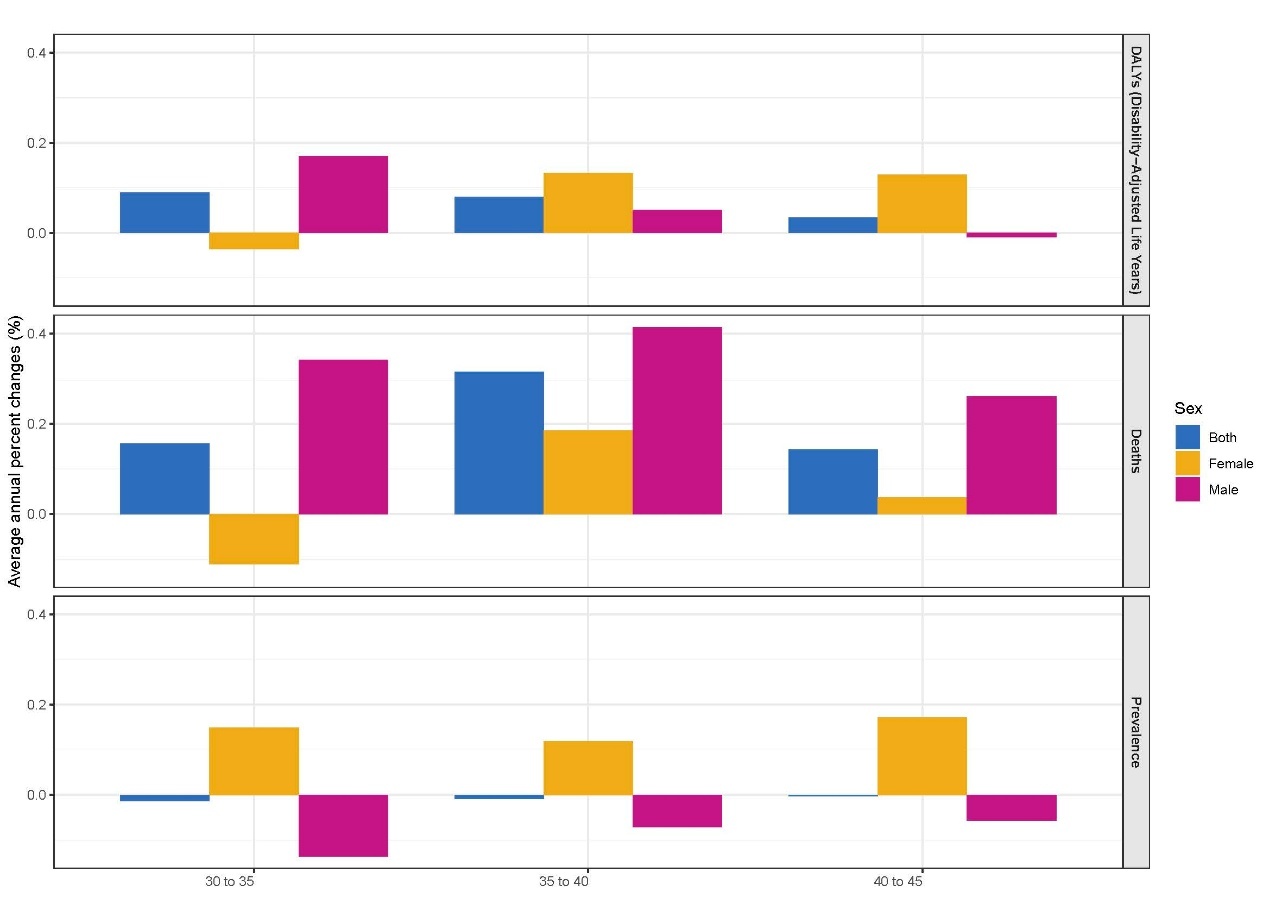


**Figure S4.** AAPC of age-standardized prevalence, ASMR and ASDR of atrial fibrillation in patients aged 30-45 years from 1990 to 2021 by sex and age.


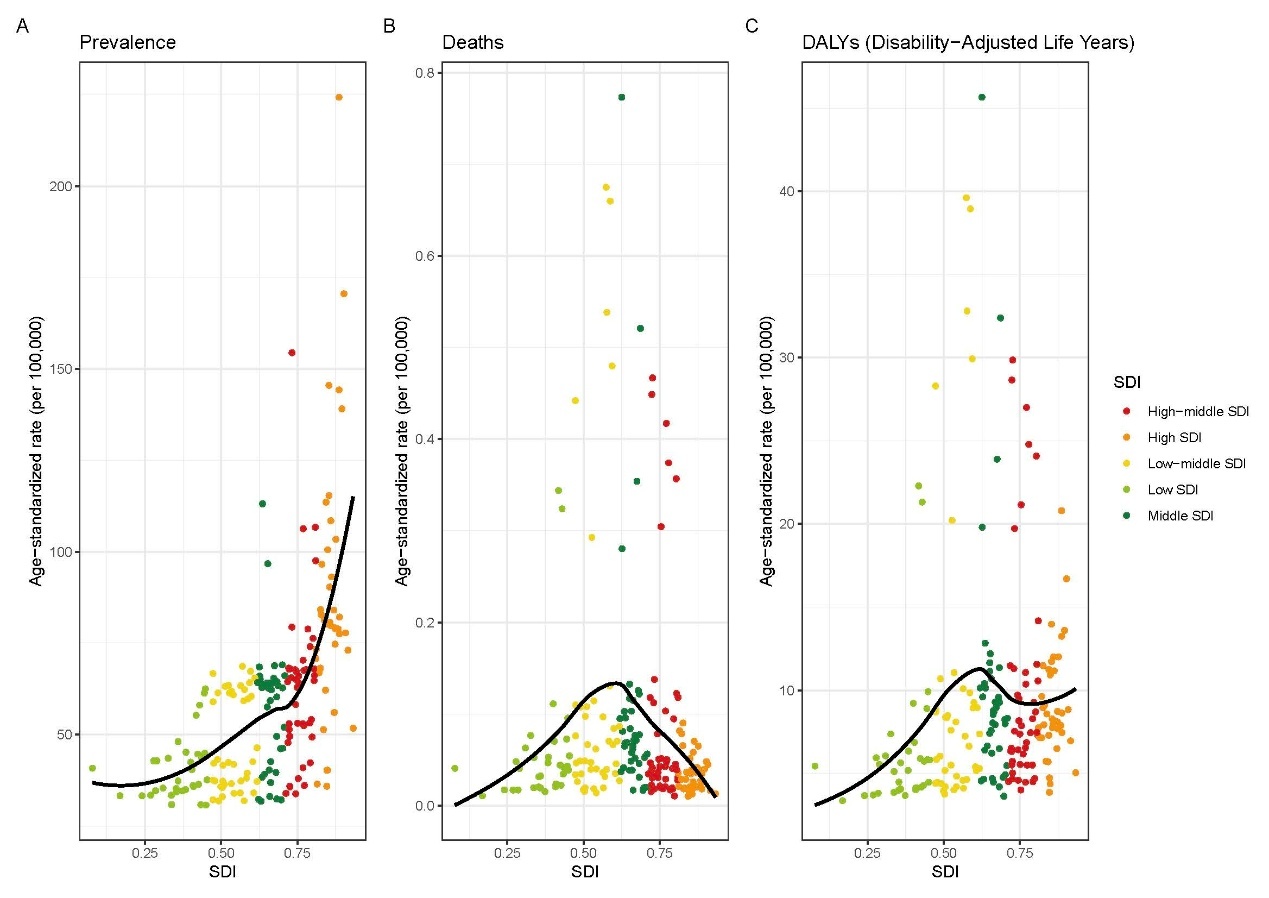


**Figure S5** Prevalence (A), mortality (B) and DALYs (C) rate of atrial fibrillation in patients aged 30-45 years from 204 countries according to the SDI in 2021.


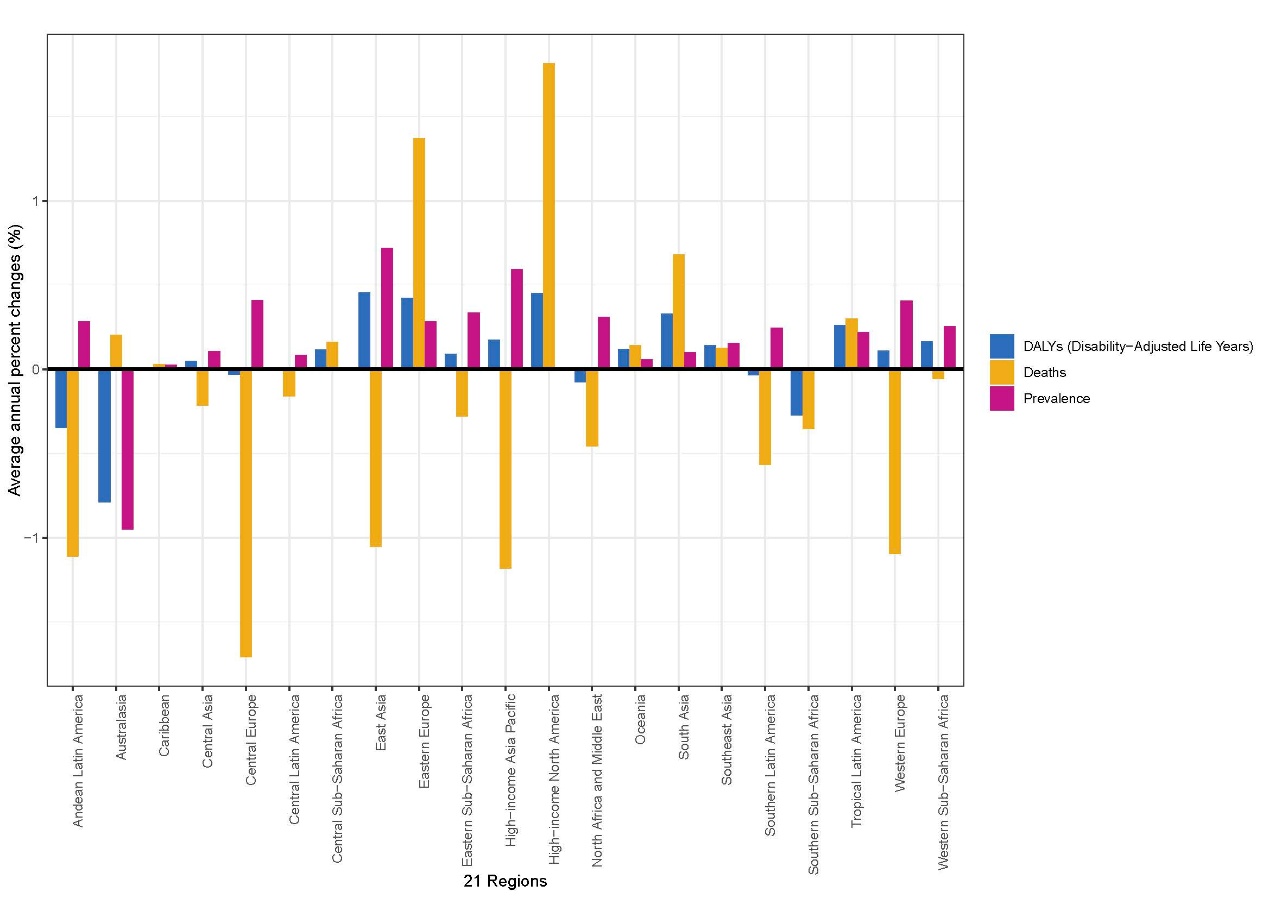


**Figure S6** AAPC of age-standardized prevalence, ASMR and ASDR of atrial fibrillation in patients aged 30-45 years from 1990 to 2021 at regional levels.


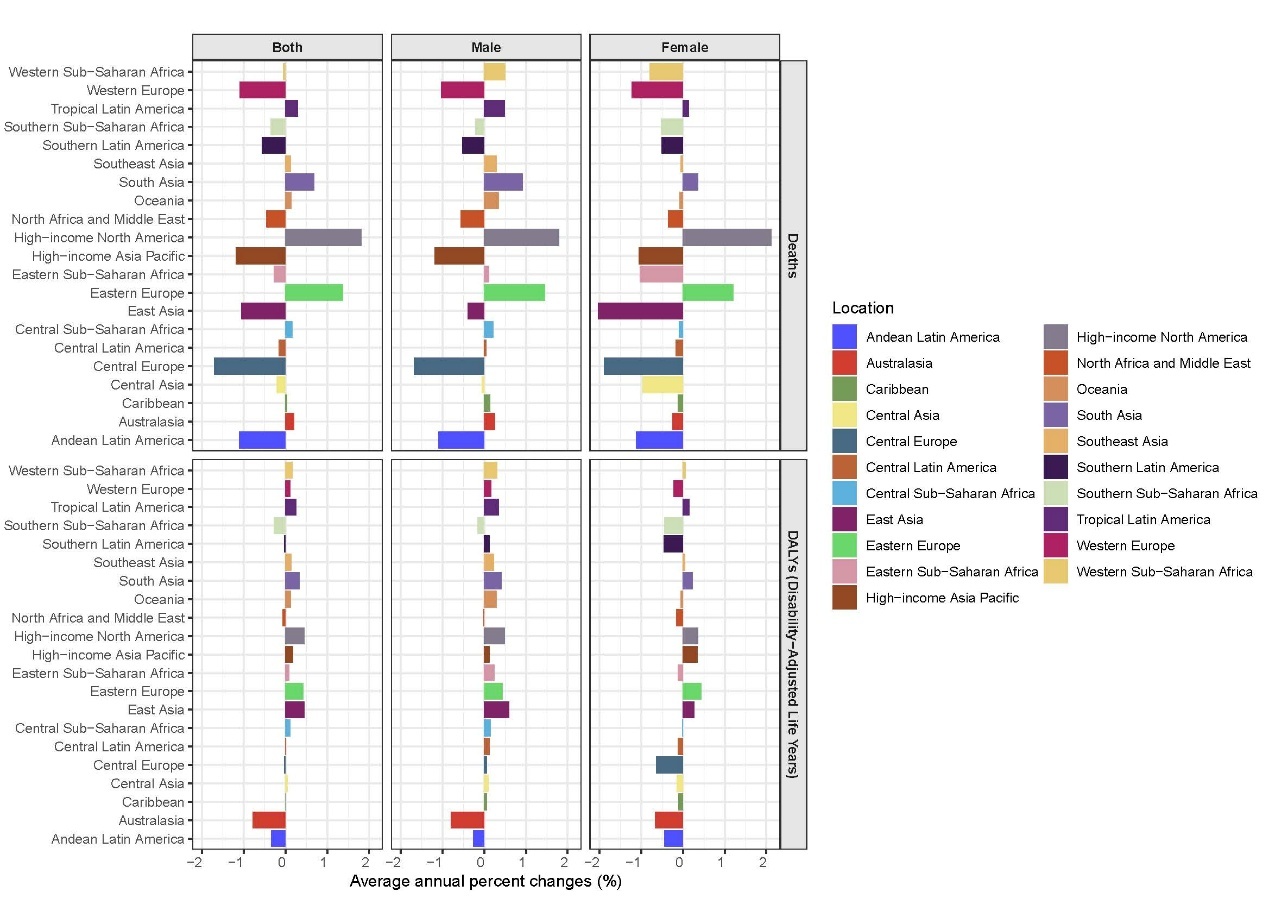


**Figure S7** AAPC of ASMR and ASDR of atrial fibrillation in patients aged 30-45 years from 1990 to 2021 at regional levels by sex.
